# Whole-transcriptome, high-throughput RNA sequence analysis of the bovine macrophage response to *Mycobacterium bovis* infection *in vitro*

**DOI:** 10.1186/1471-2164-14-230

**Published:** 2013-04-08

**Authors:** Nicolas C Nalpas, Stephen DE Park, David A Magee, Maria Taraktsoglou, John A Browne, Kevin M Conlon, Kévin Rue-Albrecht, Kate E Killick, Karsten Hokamp, Amanda J Lohan, Brendan J Loftus, Eamonn Gormley, Stephen V Gordon, David E MacHugh

**Affiliations:** 1 Animal Genomics Laboratory, UCD School of Agriculture and Food Science, University College Dublin, Belfield, Dublin, Dublin 4, Ireland; 2 UCD School of Veterinary Medicine, University College Dublin, Belfield, Dublin, Dublin 4, Ireland; 3 Smurfit Institute of Genetics, Trinity College Dublin, Trinity College, Dublin, Ireland; 4 UCD Conway Institute of Biomolecular and Biomedical Research, University College Dublin, Belfield, Dublin, Dublin 4, Ireland; 5 Tuberculosis Diagnostics and Immunology Research Centre, UCD School of Veterinary Medicine, University College Dublin, Belfield, Dublin, Dublin 4, Ireland

**Keywords:** Bovine tuberculosis, Immune response, Microarray, *Mycobacterium bovis*, Natural antisense transcript, RNA-sequencing, Transcriptome

## Abstract

**Background:**

*Mycobacterium bovis*, the causative agent of bovine tuberculosis, is an intracellular pathogen that can persist inside host macrophages during infection via a diverse range of mechanisms that subvert the host immune response. In the current study, we have analysed and compared the transcriptomes of *M. bovis*-infected monocyte-derived macrophages (MDM) purified from six Holstein-Friesian females with the transcriptomes of non-infected control MDM from the same animals over a 24 h period using strand-specific RNA sequencing (RNA-seq). In addition, we compare gene expression profiles generated using RNA-seq with those previously generated by us using the high-density Affymetrix® GeneChip® Bovine Genome Array platform from the same MDM-extracted RNA.

**Results:**

A mean of 7.2 million reads from each MDM sample mapped uniquely and unambiguously to single *Bos taurus* reference genome locations. Analysis of these mapped reads showed 2,584 genes (1,392 upregulated; 1,192 downregulated) and 757 putative natural antisense transcripts (558 upregulated; 119 downregulated) that were differentially expressed based on sense and antisense strand data, respectively (adjusted *P*-value ≤ 0.05). Of the differentially expressed genes, 694 were common to both the sense and antisense data sets, with the direction of expression (*i.e.* up- or downregulation) positively correlated for 693 genes and negatively correlated for the remaining gene. Gene ontology analysis of the differentially expressed genes revealed an enrichment of immune, apoptotic and cell signalling genes. Notably, the number of differentially expressed genes identified from RNA-seq sense strand analysis was greater than the number of differentially expressed genes detected from microarray analysis (2,584 genes versus 2,015 genes). Furthermore, our data reveal a greater dynamic range in the detection and quantification of gene transcripts for RNA-seq compared to microarray technology.

**Conclusions:**

This study highlights the value of RNA-seq in identifying novel immunomodulatory mechanisms that underlie host-mycobacterial pathogen interactions during infection, including possible complex post-transcriptional regulation of host gene expression involving antisense RNA.

## Background

Bovine tuberculosis (BTB) is caused by infection with *Mycobacterium bovis*―an intracellular pathogen belonging to the *Mycobacterium tuberculosis* complex [1-4]. BTB has major economic, animal welfare and public health consequences, and has remained recalcitrant to eradication despite the implementation of improved management strategies in recent decades [5,6]. BTB transmission is primarily caused by inhalation of infectious bacilli contained within aerosolised respiratory secretions. Following exposure, the pathogen is phagocytosed by host alveolar macrophages, which serve as key effector cells in activating the innate and adaptive immune responses required to determine the outcome of infection [7].

The immune response to *M. bovis* is similar to that elicited by *M. tuberculosis* infection in humans. Infected macrophages secrete several NF-κB-inducible inflammatory cytokines that initiate and regulate an adaptive immune response characterised by the release of IFN-γ from T-cells [8]. IFN-γ activates microbicidal activity in infected macrophages and also promotes the sequestration of the pathogen in granulomas―organised complexes of immune cells consisting of lymphocytes, non-infected macrophages, dendritic cells and neutrophils that contain mycobacterial-infected macrophages and prevent the spread of bacilli to other tissues [9-11].

In many cases, however, mycobacterial pathogens can evade the host immune response and persist within alveolar macrophages resulting in lengthy subclinical phases of infection that can lead to immunopathology and disease dissemination. Pathogen survival in alveolar macrophages is achieved through a diverse range of mechanisms including the inhibition of phagosome maturation and the suppression of key immuno-regulatory pathways that mediate the host immune response to infection [12,13]. Consequently, analysis of the macrophage transcriptome in response to *M. bovis* infection can offer a deeper understanding of the cellular processes governing pathogen-macrophage interactions and how modulation of these cellular pathways underlie progression to active BTB. Furthermore, identification of transcriptional markers of infection may enable the development of novel diagnostics for BTB, providing new tools for disease management [14,15].

The completion of an annotated *Bos taurus* reference genome sequence, together with developments in high-throughput transcriptomic technologies, such as immuno-specific and pan-genomic microarrays, have enabled detailed functional genomic investigation of the bovine host response to mycobacterial infections [16-21]. However, the recent advent of RNA sequencing (RNA-seq) technologies offers unprecedented opportunities for gene expression analysis previously unavailable for microarray technology, including unbiased whole-transcriptome profiling, the analysis of sense and antisense transcription, the characterisation of new classes of RNA, and the identification of novel mRNA splice variants [22,23]. Furthermore, the digital nature of RNA-seq data provides a more precise and sensitive method to map and quantify RNA transcripts compared to the analog data generated by microarray technologies [24].

Previously, we used the pan-genomic high-density Affymetrix® GeneChip® Bovine Genome Array to compare temporal changes in gene expression profiles in RNA extracted from *M. bovis*-infected and non-infected control bovine monocyte-derived macrophages (MDM) purified from seven age and sex-matched Holstein-Friesian females at intervals of 2, 6 and 24 h post-infection [20]. We demonstrated that the number of differentially expressed genes increased sequentially at each time point post-infection, with the highest number of differentially expressed genes observed 24 h post-infection [20].

To gain a deeper understanding of the transcriptional changes induced 24 h post-infection with *M. bovis*, we have used strand-specific RNA-seq technology for the present study, to analyse the transcriptomes of the infected and non-infected control MDM samples generated by us previously [20]. A list of differentially expressed genes was generated by comparing the MDM transcriptomes from the infected and non-infected control samples, and these genes were further analysed using the Ingenuity® Systems Pathway Analysis Knowledge Base to identify macrophage cellular pathways underlying *M. bovis* infection *in vitro*. Finally, the list of differentially expressed genes generated from analysis of the RNA-seq data was compared to results from a comparable microarray experiment published by our group [20].

## Results

### Summary statistics for the RNA-seq data

**Figure 1:**
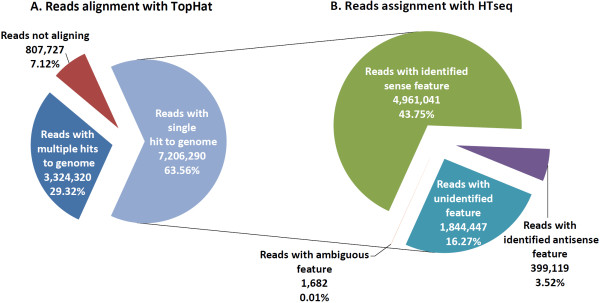
**Apportionment of reads mapping to unique and multiple locations in the *B. taurus *reference genome. A**) Pie chart showing the mean number and percentage of reads that aligned to unique location and multiple locations in the *B. taurus* reference genome using the TopHat splice junction mapper. **B**) Pie chart showing the mean number and percentage of uniquely mapped reads assigned to ambiguous gene features (*i.e.* reads that map to overlapping gene sequences), unidentified gene features (*i.e.* reads that map to the genome that have no gene annotation) and identified features (*i.e.* known gene sequences) based on sense strand and antisense strand data using the HTSeq package.

All 14 RNA-seq libraries were sequenced across seven lanes of one Illumina® flow cell with a mean of 25.5 million reads (range: 20.7 million to 29.3 million reads) generated per lane. Deconvolution and filtering of sequence reads to remove adapter-dimer sequences yielded a mean of 11.3 million reads (range: 2.7 million to 18.0 million reads) per individual RNA-seq library. Subsequent alignment of the filtered RNA-seq reads to the *B. taurus* reference genome (Btau 4.0.63 genome release) yielded a mean of 7.2 million reads (63.6%) for each RNA-seq library that mapped to unique locations in the bovine genome; a mean of 3.3 million reads (29.3%) for each library that mapped to multiple locations in the genome; and a mean of 0.8 million reads for each library (7.1%) that did not map to any genome location (Figure 1A). The number of reads per individual RNA-seq library is provided in Additional file 1: Table S1.

**Figure 2:**
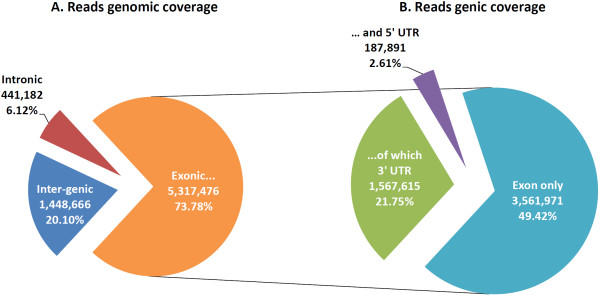
**The distribution of uniquely mapped reads.** The mean number and percentage of uniquely mapped reads are given. **A**) Pie chart showing the mean number of reads that map to inter-genic, intronic and exonic regions of the *B. taurus* reference genome. **B**) Pie chart differentiating the mean number of reads mapping to exonic regions including those that map to 5^′^-UTR- and 3^′^-UTR-associated exonic sequences.

Further analysis of the individual library reads mapping to unique locations in the *B. taurus* reference genome (7.2 million reads) revealed that a mean of 5.3 million reads (73.6%) aligned to exonic regions. 1.6 million (22.2%) and 0.2 million (0.3%) reads were associated with exon-3^′^ UTR and exon-5^′^ UTR sequences, respectively; 1.4 million reads (19.4%) mapped to intergenic locations and 0.4 million reads (5.5%) mapped to intronic regions (Figure 2). The filtered sequence reads for all deconvoluted libraries were also aligned to the complete genome sequence of the *M. bovis* AF2122/97 strain (GenBank accession number NC_002945.3) to assess the presence of mycobacterial RNA contamination in individual libraries―a mean of 234 reads (0.004%) mapped to locations in the *M. bovis* genome. The reads that mapped to the *M. bovis* genome did not map to the *B. taurus* reference genome. Following preliminary filtering and quality checks, only sequence reads that mapped to unique locations in the *B. taurus* reference genome were used for downstream bioinformatics and IPA analyses.

Quantification of the number of reads that exclusively mapped to genes with bovine Ensembl IDs was performed using the HTseq-count software package. This analysis demonstrated that a mean of 5.0 million reads (43.8%) per library mapped to Ensembl gene IDs based on sense strand sequence information, with a mean of 0.4 million sequence reads for each library (3.5%) mapping to Ensembl gene IDs based on antisense strand sequence information; only these two sets of reads were used to separately derive gene expression values for sense and antisense strand transcription, respectively. Of the remaining reads—which were not used to derive gene expression values—a mean of 1,600 reads (0.01%) for each library were associated with multiple Ensembl gene IDs, while a mean of 1.8 million reads for each library (16.3%) were not associated with Ensembl gene IDs (Figure 1B).

Prior to multi-dimensional scaling (MDS) and differential gene expression analysis, density plots (Additional file 2: Figure S1) displaying the number of sequence reads per gene were constructed and analysed. Two RNA-seq libraries comprising a control and *M. bovis*-infected sample from the same animal (animal number 700; Additional file 1: Table S1) showed skewed distributions compared to all other libraries; consequently these samples were removed from all further downstream analyses. All downstream analyses, including differential gene expression, IPA analysis based on sense and antisense strand data, and technical comparisons between RNA-seq and microarray platforms were performed using data from the remaining six animals (*i.e.* 12 RNA-seq libraries).

### Gene expression and IPA analysis of sense strand transcription

Analysis of the gene coverage using reads mapping to unique locations of the *B. taurus* reference genome based exclusively on sense strand sequence information, showed that of the 25,669 annotated *B. taurus* genes, 15,422 genes (60.1%) had at least one sequence read count (*i.e.* one mapped read) in at least one library. The 15,422 detectable genes were further filtered by removing lowly expressed genes. Consequently, only genes displaying more than one count per million reads for at least three libraries were used for subsequent analyses. This yielded 11,131 genes (43.4% of annotated *B. taurus* genes) that were suitable for downstream analyses.

**Figure 3:**
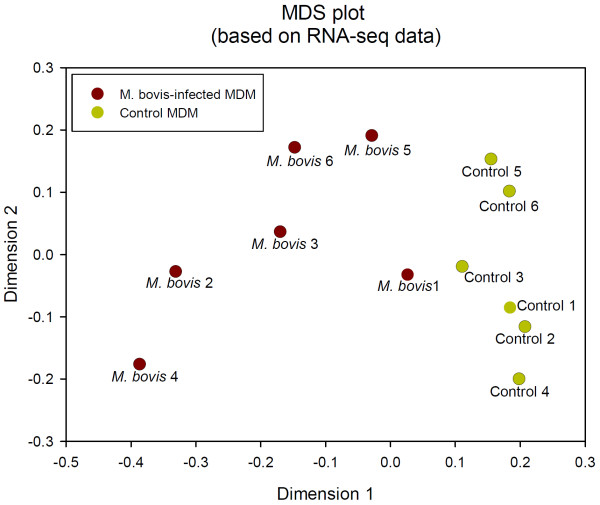
**Multi-dimensional scale plot of all *M. bovis*-infected and control samples based on RNA-seq sense data.** Dimension 1 and dimension 2 separate all 12 RNA-seq libraries based on the expression value of the 11,131 genes (based on RNA-seq sense strand data only) that passed all data filtering criteria prior to differential gene expression analysis.

Prior to differential gene expression analysis, the data from the 11,131 filtered genes were used for MDS analysis (Figure 3). MDS analysis demonstrated that MDM samples were differentiated according to treatment status (*i.e.* infected versus non-infected control samples) along dimension 1, while dimension 2 separated the MDM samples according to animal ID.

Statistical analysis of all 11,131 genes that passed the filtering process identified a total of 2,584 differentially expressed genes (adjusted *P*-value ≤ 0.05), of which 1,392 were upregulated and 1,192 were downregulated in the *M. bovis*-infected MDM relative to the non-infected control MDM. In addition, expression fold-change values were markedly higher for upregulated genes compared to downregulated genes. A list of the differentially expressed genes based on sense strand data is presented in Additional file 3: Table S2.

Among the most upregulated genes based on expression fold-change (Table 1), were the adrenergic, beta-3-, receptor gene [*ADRB3*] (2^nd^ ranked upregulated gene); the interleukin 17A gene [*IL17A*] (3^rd^ ranked); the serum amyloid A 3 gene [*SAA3*] (4^th^ ranked); and the mammary serum amyloid A3.2 gene [*M-SAA3.2*] (5^th^ ranked). The first ranked upregulated gene is not described here as it encodes an uncharacterized protein. The most downregulated genes were the gamma-aminobutyric acid (GABA) B receptor, 2 gene [*GABBR2*] (1^st^ ranked downregulated gene); the cadherin 26 gene [*CDH26*] (2^nd^ ranked); the solute carrier family 16, member 12 (monocarboxylic acid transporter 12) gene [*SLC16A12*] (3^rd^ ranked); the Fas apoptotic inhibitory molecule 2 gene [*FAIM2*] (4^th^ ranked); and the SH3 domain and tetratricopeptide repeats 2 gene [*SH3TC2*] (5^th^ ranked).

**Table 1:** List of the top five ranking up- and downregulated genes based on the RNA-seq sense strand data

**Rank**	**Direction of expression**	**Ensembl Gene ID**	**Gene symbol**	**Gene name**	**Log**_**2**_**fold change**	**Adjusted*P*-value**
1	Upregulated	ENSBTAG00000023064	Not available	Uncharacterized protein	8.41	7.62 × 10^-21^
2	Upregulated	ENSBTAG00000017981	*ADRB3*	adrenergic, beta-3-, receptor	8.01	2.48 × 10^-17^
3	Upregulated	ENSBTAG00000002150	*IL17A*	interleukin 17A	7.71	1.37 × 10^-14^
4	Upregulated	ENSBTAG00000022396	*SAA3*	serum amyloid A 3	7.50	7.67 × 10^-114^
5	Upregulated	ENSBTAG00000010433	*M-SAA3.2*	mammary serum amyloid A3.2	6.95	5.26 × 10^-41^
1	Downregulated	ENSBTAG00000013810	*GABBR2*	gamma-aminobutyric acid (GABA) B receptor, 2	−6.67	5.49 × 10^-08^
2	Downregulated	ENSBTAG00000020261	*CDH26*	cadherin 26	−5.68	4.25 × 10^-18^
3	Downregulated	ENSBTAG00000004662	*SLC16A12*	solute carrier family 16, member 12 (monocarboxylic acid transporter 12)	−5.52	3.01 × 10^-16^
4	Downregulated	ENSBTAG00000017504	*FAIM2*	Fas apoptotic inhibitory molecule 2	−4.40	6.53 × 10^-12^
5	Downregulated	ENSBTAG00000017151	*SH3TC2*	SH3 domain and tetratricopeptide repeats 2	−4.28	4.29 × 10^-10^

List of the top most upregulated and downregulated genes in *M. bovis*-infected samples relative to the control samples are shown (ranked by fold change) based on RNA-seq sense strand data.

**Figure 4:**
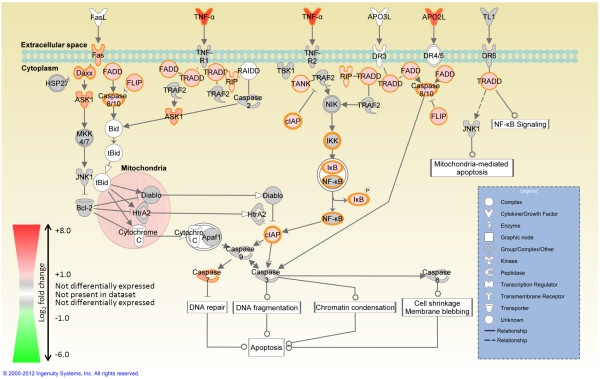
**Pathway for *Death receptor signalling *at 24 h post-*M. bovis *infection.** Genes associated with *Death receptor signalling* canonical pathway that show differential expression are highlighted in colour. Colour intensity indicates the degree of upregulation (red) or downregulation (green) relative to the control MDM samples. Grey shading indicates genes that were not significantly differentially expressed; white shading represents genes in the pathway which did not pass the filtering for differential expression analysis.

Functional categorisation of the 2,584 differentially expressed genes using IPA revealed an enrichment of genes with roles in immunology, cell development and proliferation, and apoptosis (Additional file 4: Table S3). IPA was also used to identify canonical pathways enriched for differentially expressed genes, many of which were had immune and apoptotic functions. (Additional file 5: Table S4). Based on the well-documented role of apoptosis in the host response to mycobacterial infection [25] the *Death receptor signalling* canonical pathway was overlaid with RNA-seq gene expression results and is presented in Figure 4.

### Gene expression analysis of antisense strand read data

Analysis of the RNA-seq reads that mapped to antisense strand gene sequences revealed that 13,796 genes of the 25,669 annotated *B. taurus* genes had at least one sequence read count (*i.e.* one mapped read count) for at least one library. Filtering out of lowly expressed genes (as detailed for the sense strand data above) led to the exclusion of 6,954 genes, leaving a total of 6,842 genes for differential expression analysis based of antisense strand data. 757 of the genes were differentially expressed (adjusted *P*-value ≤ 0.05), of which 558 showed increased expression and 199 showed decreased expression in the *M. bovis*-infected MDM relative to the non-infected control MDM. As with the sense strand data, the fold-change in expression for the genes showing increased expression was markedly higher than that for the genes decreasing in expression. A list of significant differentially expressed genes derived from the antisense strand expression data is presented in Additional file 6: Table S5. Additional file 7: Figure S2 shows the distribution of sense and antisense reads that mapped to the spectrin, beta, erythrocytic gene (*SPTB*). As these reads do not map to the exact same locations in the *SPTB* gene, we are confident that they represent actual antisense transcripts based on the current annotation of the *B. taurus* reference genome and are not due to technical artefacts introduced during RNA-seq library preparation.

**Figure 5:**
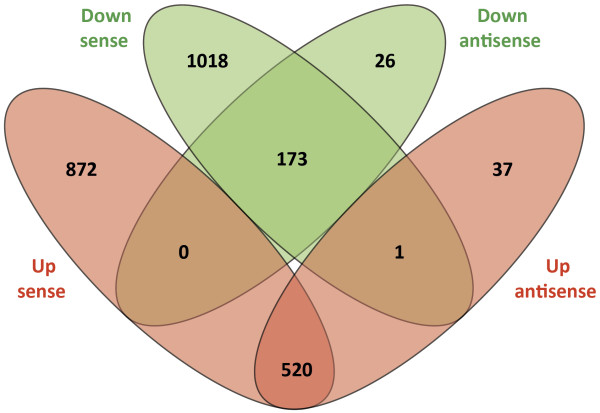
**Venn diagrams showing comparison of RNA-seq sense versus antisense strand differential gene expression.** Venn diagram showing the comparison of the direction of differential expression based on sense and antisense strand data. Upregulated and downregulated genes are shaded red and green, respectively.

A systematic comparison was performed between significantly differentially expressed genes detected using the sense strand RNA-seq data (*n* = 2,584) and those identified from the antisense strand data (*n* = 757). This generated a list of 694 differentially expressed genes that were common to both the sense strand and the antisense strand data sets. The common 694 genes were further subdivided according to the direction of expression (*i.e.* up- or downregulated) in the *M. bovis*-infected MDM relative to the control MDM. 520 genes displayed an increase in expression in both the sense and antisense strand data sets, while 173 genes showed decreased expression from both strands. In addition, no gene displayed increased expression from the sense strand and decrease expression from the antisense strand. Finally, one gene showed decreased expression from the sense strand and increased expression from the antisense strand (Figure 5).

Functional categorisation of the 757 differentially expressed genes based on antisense strand data using IPA revealed an enrichment of genes with roles in immunology, cell development and proliferation, and response to infection (Additional file 8: Table S6). Four of the top five ranking GO categories identified from the antisense and sense strand data were identical (Additional file 4: Table S3 and Additional file 8: Table S6). Similarly, IPA analysis of the antisense strand data generated a list of canonical pathways with immune related function (Additional file 9: Table S7). Four of the five top-ranking canonical pathways identified from the antisense and sense strand data were identical (Additional file 5: Table S4 and Additional file 9: Table S7).

### Comparison of differential gene expression profiles obtained from RNA-seq and microarray platforms

The total RNA samples extracted from *M. bovis*-infected and non-infected control MDM samples described here, were previously used by us for gene expression profiling with the Affymetrix® GeneChip® Bovine Genome Array [20]. This array contains 24,027 probe sets representing more than 23,000 transcripts and includes approximately 19,000 UniGene clusters [26].

In order to directly compare gene expression profiles from these previously generated microarray data with the RNA-seq data generated in the current study, a number of steps were performed. Firstly, we removed the same two samples which were excluded from the RNA-seq data analysis (*i.e.* the non-infected control and *M. bovis*-infected MDM-extracted RNA from animal number 700), then re-analysed all microarray data using all 12 RNA samples from the remaining six animals. Secondly, we mapped Affymetrix® GeneChip® Bovine Genome Array probes to genes with bovine Ensembl gene IDs based on data from the *B. taurus* reference genome. Of the 24,072 probe sets represented on the array, 11,790 probe sets passed filtering; with 8,560 unique probe sets mapping to a *B. taurus* Ensembl gene ID, representing 6,807 unique genes. Prior to differential gene expression analysis, the data from the 11,790 filtered probes was used for MDS analysis (Additional file 10: Figure S3). As with the data from the RNA-seq, the MDS analysis demonstrated that MDM samples were differentiated according to treatment status (*i.e.* infected versus non-infected control samples) along dimension 1, while dimension 2 separated the MDM samples according to animal ID. It is important to note that joint inspection of Figure 3 and Additional file 10: Figure S3 shows that the samples are spatially arranged in a very similar pattern for the two different gene expression platforms.

#### Comparison of the RNA-seq and microarray dynamic ranges

To assess the dynamic range of the RNA-seq and microarray data sets, we analysed the log_2_ microarray intensities and the log_2_ reads per kilobase per million mapped reads (RPKM) values for all genes that passed the filtering criteria. From this, we calculated the dynamic range of the microarray by subtracting the gene with the highest mean log_2_ intensity (ENSBTAG00000018784 [*CTSZ*]; log_2_ intensity = 15.48) from the gene with the lowest mean log_2_ intensity (ENSBTAG00000002021 [*BNIP1*]; log_2_ intensity = 2.03), yielding a log_2_ dynamic range of 13.45. We then calculated the dynamic range of RNA-seq by subtracting the gene with the highest mean log_2_ RPKM (ENSBTAG00000043561 [*COX1*]; log_2_ RPKM =12.60) from the gene with the lowest mean log_2_ RPKM (ENSBTAG00000001325 [*UPB1*]; log_2_ RPKM = −6.83), yielding a log_2_ dynamic range of 19.43. Our analysis therefore shows that the dynamic range for RNA-seq is greater than the microarray. This method for dynamic range calculations of gene expression data has been previously described by Chen and colleagues [27]. Furthermore, it is important to note that raw RPKM values are proportions; consequently, values less than 1.0 yield negative values when log_2_-transformed.

To ensure that differences in the dynamic ranges were not due to contrasting normalisation procedures, we determined the dynamic range of the RNA-seq data following a quantile normalisation strategy, which is comparable to the quantile normalisation used for the microarray. This analysis yielded a log_2_ dynamic range for the RNA-seq data of 20.01, which further supports a greater dynamic range for the RNA-seq platform compared to the microarray.

#### Comparison of high and low transcript expression from RNA-seq and microarray data

For this analysis, we compared log_2_ reads per kilobase (of transcript sequence) per million reads (RPKM) with the log_2_ raw microarray hybridisation intensity probe signals. Genes with alternative transcripts—for which, a definitive gene length necessary for RPKM calculations could not be derived—were omitted from this analysis. Spearman rank correlation analysis was first performed for a total of 5,560 common transcripts that passed the RNA-seq and microarray filtering criteria (detailed in Methods). This analysis was performed separately for the non-infected control and infected MDM samples. We observed a significant (*P* ≤ 0.01) Spearman correlation coefficient of 0.66 and 0.64 for the infected and the control samples, respectively. These results suggest that there is a positive relationship between the microarray and RNA-seq data sets such that: (1) transcripts that yield a high intensity value on the microarray also yield a high read count based on the RNA-seq platform; and (2) transcripts that yield a low intensity value on the microarray also yield a low read count based on the RNA-seq platform.

We next binned the transcripts into groups of highly, moderately and lowly expressed transcripts (based on the microarray data), with an approximately equal number of transcripts in each bin. We observed the greatest correlation between the highly expressed transcripts (*r* = 0.551 for infected MDM; *r* = 0.535 for control MDM; *P* ≤ 0.01), followed by the lowly expressed transcripts (*r* = 0.302 for infected MDM; *r* = 0.327 for control MDM; *P* ≤ 0.01) followed by the moderately expressed transcripts (*r* = 0.261 for infected MDM; *r* = 0.217 for control MDM; *P* ≤ 0.01). These results suggest that the correlation between the two platforms is better for highly abundant transcripts compared to moderately and lowly abundant transcripts.

#### Comparison of expression fold-change and difference in expression value between treatments for RNA-seq and microarray data

To investigate if the greatest fold-change in expression was largely observed in genes with low levels of expression, we compared: (1) the log_2_ fold-change with the log_2_ differences in counts per million reads (CPM) between the control and infected MDM groups for the RNA-seq platform; and (2) the log_2_ fold-change with the log_2_ differences in hybridisation intensity between the control and infected MDM groups for the microarray platform. We hypothesised that if transcripts with the lowest expression gave the highest fold-change values, then a negative correlation between log_2_ fold-change and log_2_ differences for genes displaying increased expression post-infection. Reciprocally, a positive correlation would be expected between log_2_ fold-change and log_2_ differences for genes displaying decreased expression post-infection.

For RNA-seq, this analysis was based on 11,131 genes that passed the filtering criteria; of which, 5,377 displayed increased expression and 5,754 genes displayed decreased expression following MDM infection. We observed Spearman correlation coefficients of 0.536 and −0.394 for the genes displaying increased and decreased expression following infection, respectively (*P* ≤ 0.001). These correlation coefficients contrast with those expected based on our hypothesis described above; therefore, we conclude that there is no obvious relationship between gene expression level and fold-change.

For the microarray, our analysis was based on 11,665 genes that passed the filtering criteria; of which, 5,020 displayed increased expression and 6,645 genes displayed decreased expression following MDM infection. Spearman correlation coefficients of 0.378 and −0.128 were observed for the genes displaying increased and decreased expression following infection, respectively (*P* ≤ 0.001). Again, we conclude that there is no obvious relationship between gene expression level and fold-change.

#### Comparison of fold-change in expression from RNA-seq and microarray data

We compared log_2_ fold-changes in expression for a total of 6,183 genes common to both RNA-seq and the microarray that passed the data filtering criteria. This analysis encompassed, but was not restricted, to the differentially expressed genes identified from both data sets, the description and analysis of which is presented below. Note the number of genes analysed here was greater than the total number of transcripts analysed for transcript expression comparison as RPKM could not be calculated for genes with alternative transcripts as stated above (so reducing the number of comparable genes in the analysis above). The Spearman correlation coefficient of log_2_ fold-changes between the RNA-seq and microarray was 0.858 (*P* ≤ 0.01), indicating that the fold-change magnitudes for the platforms were very similar.

#### Comparison of the number and fold-change of differentially expressed genes identified from RNA-seq and microarray

**Figure 6:**
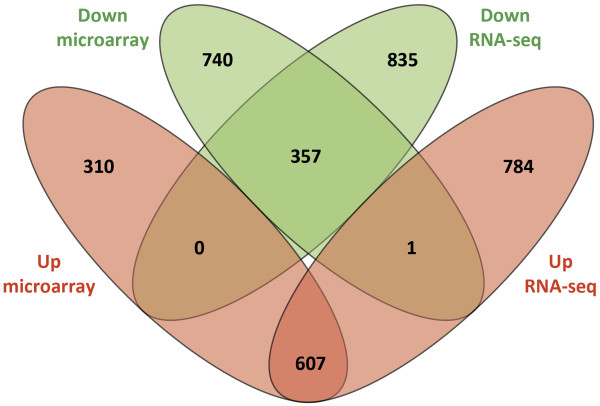
**Venn diagram showing comparison of differentially expressed genes identified from alternative transcriptomic platforms.** Venn diagram showing overlap of the upregulated and downregulated genes as identified with microarray and RNA-seq platforms. Sets of upregulated genes are represented in red, sets of downregulated genes are in green.

Subsequent analysis of the microarray data showed that 2,015 unique genes were differentially expressed, with 917 genes and 1,098 genes displaying up- and downregulation, respectively, in the infected MDM relative to the control MDM (FDR ≤ 0.05; Additional file 11: Table S8). Comparison of the differentially expressed genes with Ensembl gene IDs identified from the microarray and RNA-seq sense strand data sets revealed 964 differentially expressed genes displaying the same direction of expression that were common to both the microarray and RNA-seq data sets; these comprised 607 upregulated and 357 downregulated common genes. One gene was found respectively downregulated and upregulated in the microarray and RNA-seq sense strand data sets (Additional file 12: Table S9). Of the remaining differentially expressed genes detected, 1,050 genes (comprising 310 upregulated genes and 740 downregulated genes) were unique to the microarray data set, while 1,619 genes (comprising 784 upregulated genes and 835 downregulated genes) were unique to the RNA-seq data set (Figure 6). The differentially expressed genes that were common to both platforms and displayed the same direction of expression, represent 47.8% (*i.e.* 964/2,015 genes) and 37.3% (*i.e.* 964/2,584 genes) of all differentially expressed genes detected by microarray and RNA-seq analysis, respectively.

Spearman rank analysis of the log_2_ RPKM values (RNA-seq data) with the log_2_ hybridisation intensity signals (microarray data) for all 964 differentially expressed genes that were common to both platforms and had the same direction of expression revealed a correlation coefficient of *r* = 0.713 for infected MDM and *r* = 0.660 for control MDM (*P* ≤ 0.01), indicating that there is a positive relationship between raw signal intensities for each platform/treatment group. Finally, comparison of the log_2_ fold-change in gene expression between the two platforms for these 964 genes yielded a correlation coefficient of *r* = 0.929 (*P* ≤ 0.001).

To investigate the effect of platform dynamic range on the number differentially expressed genes unique to each platform we plotted and compared the density of the log_2_ of the mean hybridisation intensity and the density of the log_2_ of the mean CPM for the differentially expressed genes unique to each platform and for the differentially expressed genes common to both platforms. This analysis showed that differentially expressed genes uniquely detected by RNA-seq are largely characterised by low levels of expression for both the control and infected MDM groups. This pattern was not observed for the microarray data (Additional file 13: Figure S4). This analysis shows that the sensitivity of RNA-seq for the detection of lowly expressed transcripts is greater than the microarray.

### IPA analysis of differentially expressed genes common to both microarray and RNA-seq

To validate the IPA analysis performed using differentially expressed genes based on RNA-seq sense strand data only, we uploaded the 965 differentially expressed genes common to both RNA-seq and the microarray into IPA (964 genes plus the one gene that displayed reciprocal expression for the two platforms). We hypothesised that the 965 common genes were more likely to reflect true biological changes induced following infection with *M. bovis*, compared to the differentially expressed genes identified by either platform independently. This approach has been previously used to validate GO category analysis in a study where RNA-seq and microarray data were available for the same samples [28]. Of the 63 GO categories identified for the common differentially expressed genes, 56 (88.9%) and 60 (95.2%) were observed in the RNA-seq and microarray IPA data sets, respectively (Additional file 4: Table S3, Additional file 14: Table S10 and Additional file 15: Table S11). Similarly, of the 198 canonical pathways identified for the common differentially expressed genes, 160 (80.8%) and 166 (83.8%) were observed in the RNA-seq and microarray IPA data sets, respectively (Additional file 5: Table S4, Additional file 16: Table S12 and Additional file 17: Table S13). This analysis suggests that meaningful biological interpretation of data from either RNA-seq or microarrays is possible.

### Validation of differentially expressed genes using real time quantitative reverse transcription PCR

For method validation purposes, we quantified a panel of 16 genes via real time quantitative reverse transcription PCR (qRT-PCR) using the same MDM-extracted RNA samples that were used for the RNA-seq and microarray platforms as described above. The methods used for real time qRT-PCR analysis of these genes have been described by us elsewhere [20]. Using these data, we estimate the dynamic range of the real time qRT-PCR platform has a log_2_ dynamic range of 15—this is based on C_t_ value ranges between 20 and 35 for a number of cytokine genes.

Real time qRT-PCR analysis showed that 12 of these genes (*CCL4*, *CCL5*, *CCL20*, *CD40*, *CFB*, *CXCL2*, *IL15*, *IL1B*, *IL6*, *IRF1*, *NFKB2* and *TNF*) were upregulated, while two genes (*AREGB* and *FOS*) were downregulated in the infected MDM (*P* ≤ 0.05). The remaining two genes, *PIK3IP1* and *SPRY2* were not differentially expressed based on analysis of the real time qRT-PCR data.

We compared the real time qRT-PCR expression profiles of these 16 genes with their expression profiles from RNA-seq and the microarray data based on the analyses performed in the current study. Ten of the 12 upregulated genes (as determined by real time qRT-PCR analysis) were also upregulated in the RNA-seq and microarray platforms―only the *IL15* and *IRF1* genes were not differentially expressed based on microarray and RNA-seq results, respectively. Two genes (*AREGB* and *FOS*) were shown to be downregulated across all three gene expression technologies, while the remaining two genes (*PIK3IP1* and *SPRY2*) were shown to be downregulated based on results generated from microarray and RNA-seq only. Pairwise comparisons of the analytical platforms used across all 16 genes yielded concordances of 87.50% for the microarray and RNA-seq comparison, 81.25% for the real time qRT-PCR and RNA-seq comparison and 81.25% for the microarray and the real time qRT-PCR. This is summarised in Table 2.

**Table 2:** Comparison of fold-changes in gene expression based on RNA-seq, microarray and real time qRT-PCR results

**Gene symbol**	**Gene name**	**Gene description**	**RNA-seq fold-change**	**Array fold-change**	**Real time qRT-PCR fold-change**
*CCL4*	Chemokine (C-C motif) ligand 4	A proinflammatory and chemotactic chemokine	40.02	10.53	26.51
*CCL5*	Chemokine (C-C motif) ligand 5	A proinflammatory chemokine involved in the chemotaxis of monocytes and T-helper cells	17.43	14.68	19.12
*CCL20*	Chemokine (C-C motif) ligand 20	A chemokine involved in the chemoattraction of lymphocytes and neutrophils	67.34	82.33	45.94
*CD40*	CD40 molecule, TNF receptor superfamily member 5	A member of the TNF-receptor superfamily. Mediates the immune and inflammatory responses	13.43	9.99	12.32
*CFB*	Complement factor B	A component of the alternative pathway of complement activation	3.87	40.63	40.36
*CXCL2*	Chemokine (C-X-C motif) ligand 2	An immunoregulatory chemokine produced by activated monocytes and neutrophils at sites of inflammation	15.72	4.44	14.12
*IL15*	Interleukin 15	A cytokine that regulates T and natural killer cell activation and proliferation	3.07	Not DE	2.23
*IL1B*	Interleukin 1, beta	A cytokine that mediates the inflammatory response including cell proliferation, differentiation and apoptosis	34.84	92.28	41.21
*IL6*	Interleukin 6	A cytokine that functions in inflammation and the maturation of B cells	39.62	88.47	55.08
*IRF1*	Interferon regulatory factor 1	A member of the interferon regulatory transcription factor family. An activator of interferon alpha and beta transcription	Not DE	10.01	9.74
*NFKB2*	Nuclear factor of kappa light polypeptide gene enhancer in B-cells 2 (p49/p100)	A pleiotropic transcription factor involved in inflammation, immunity, differentiation, cell growth and apoptosis	5.35	8.68	5.76
*TNF*	Tumor necrosis factor (TNF superfamily, member 2)	A proinflammatory cytokine (secreted by macrophages) involved in the regulation cell proliferation, differentiation and apoptosis, and coagulation.	15.16	80.07	15.00
*AREGB*	Amphiregulin B	A growth-modulating glycoprotein	−3.84	−9.70	−5.09
*FOS*	FBJ murine osteosarcoma viral oncogene homolog	A leucine zipper protein member of the AP-1 transcription factor complex.	−3.69	−5.50	−3.61
*PIK3IP1*	Phosphoinositide-3-kinase interacting protein 1	Suppresses the activity of phosphatidylinositol-3-kinase (PI3K), a regulator of cell division	−2.06	−1.68	Not DE
*SPRY2*	Sprouty homolog 2 (*Drosophila*)	An inhibitor of receptor tyrosine kinase signalling proteins	−2.12	−4.64	Not DE

Geometric mean fold-changes of gene expression (*M. bovis*-infected samples relative to control samples) are given for the RNA-seq sense strand, microarray and real time qRT-PCR data. Unless specified, results for each gene using the three techniques displayed significant differential expression (*P*-value ≤ 0.05). ‘Not DE’ indicates that a gene was not differentially expressed.

## Discussion

Analysis of the host transcriptome in response to mycobacterial infection has greatly enhanced our knowledge of the immunological mechanisms and cellular pathways that underlie initial infection, disease progression and ultimately active disease. These investigations have been underpinned by continual improvements in the technologies used for functional genomics and the bioinformatics methods for data analysis—particularly for microarray technologies, which have enabled analyses of immune-specific and pan-genomic gene expression patterns following infection [15,17-21,29]. Despite notable progress in understanding the molecular basis of host-mycobacteria interactions during infection, microarrays are not without their limitations, including: (1) a requirement for prior DNA sequence knowledge of annotated genes for probe design; (2) indirect quantification of gene expression by hybridisation signal intensities; (3) constrained dynamic ranges that impair quantification of lowly and highly expressed gene transcripts; and (4) a limited ability to detect splice variants and novel classes of non-coding regulatory RNA [30-32].

The RNA-seq approach, which is based on ultra-high throughput sequencing of total RNA and systematic counts of all expressed transcripts, has the potential to overcome many of the limitations associated with microarray technology. In particular, RNA-seq: (1) requires no prior sequence information (however, a reference genome is normally used, but not required for mapping of raw sequence reads) [33]; (2) has a larger dynamic range and is more sensitive than microarray data because the quantification of each gene transcript is based directly on the number of reads mapping to a particular gene; and (3) may provide additional information regarding the complexity of the transcriptome, including strand-specific gene expression, identification of novel genes, and identification of splicing events and a wide range of non-coding RNA species [23,34]. Therefore, RNA-seq can provide a sensitive, unbiased and fully quantitative and qualitative transcriptomic profile of host cells following mycobacterial infection. Consequently, we have used strand-specific RNA-seq to examine the transcriptome of bovine MDM following a 24 h *in vitro* infection with *M. bovis* (multiplicity of infection [MOI] 2:1) to gain novel insights into the transcriptional changes and cellular pathways induced during the early stages of infection. In addition, as the MDM samples described here have been previously studied using the Affymetrix® GeneChip® Bovine Genome Array, we have performed a comparison of the results generated from both analytical platforms.

### Summary of RNA-seq results

For each individual RNA-seq library analysed here, we obtained a mean of 7.2 million 69 bp reads that mapped to single unique locations in the *B. taurus* reference genome. This yielded a mean of 496.8 Mb of sequence per individual library. Examination of the gene coverage from these uniquely mapping reads demonstrated that 15,422 genes (based only on sense strand data) from a total of 25,669 annotated bovine genes (60.1%) gave at least one sequence read count in total. However, studies have shown that gene expression analysis using RNA-seq is dependent on sequencing depth and that genes with low sequencing coverage are more susceptible to the generation of false-positive differentially expressed genes. In this regard, we only considered genes displaying more than one count per million reads in at least three libraries for differential gene expression analysis; consequently a total of 11,131 genes (43.4% of the total bovine gene content) based on sense strand data were used in the analysis presented here. Although this stringent threshold lowers the number of genes for which differential expression can be performed, we believe that this criterion is sufficient for quantification and analysis of highly expressed genes with a corresponding reduction in the number of Type I errors due to lowly expressed genes [35-37].

### Sense strand gene expression and IPA analyses

Gene expression analysis of the sense strand information in the current study detected a total of 2,584 differentially expressed genes (adjusted *P*-value ≤ 0.05), of which 1,392 and 1,192 were upregulated and downregulated, respectively, in the *M. bovis*-infected MDM relative to the non-infected control MDM. This finding contrasts with previous work performed by us and others showing that mycobacterial infection *in vitro* and *in vivo* results in a higher number of downregulated genes relative to upregulated genes based on microarray analysis [17,20,21,29,38]. This discrepancy is most obviously explained by differences in sensitivity and technical biases between the two transcriptomic platforms used. This assertion is supported in the current study by the increased dynamic range of RNA-seq compared to the microarray platform. In agreement with previous studies, however, the expression fold-change values were markedly higher for upregulated genes compared to downregulated genes [17,19-21].

Analyses performed at the gene level revealed that all the top upregulated and downregulated genes have roles and functions involved in or related to immune response and infectious disease. One of the most highly upregulated genes identified was *ADRB3*; the protein encoded by this gene has been shown to have functions in carbohydrate metabolism, energy reserve metabolism, positive regulation of the MAPK cascade and regulation of apoptosis [39,40]. *IL17A* was also upregulated and has known role in the pro-inflammatory response, expansion and recruitment of innate immune cells, production of defensins and antimicrobial peptides, and linking innate and adaptive immune responses [41]. Also upregulated was *SAA3*, which was the most statistically significant gene in the data set, and has a major multifunctional role in the acute-phase response [42].

Among the most highly downregulated genes was *GABBR2*, which has multiple roles and functions, including involvement in pulmonary disorder and regulation of the coughing process [43-45]. In addition, *CDH26* was downregulated and cadherins have been shown to have a role in Ca^2+^-dependent cell-to-cell adhesion as well as cell polarisation and cell migration [46]; however, the specific role of *CDH26* has not been characterised. The *SLC16A12* gene was also detected, a member of a gene family containing genes recently shown to be associated with tuberculosis susceptibility in cattle [47]. *FAIM2* was highly downregulated, a gene with a role in the inhibition of apoptosis, a key process associated with mycobacterial infection of macrophages [25,48]. Finally, *SH3TC2* was downregulated, which interacts with members of the RAB11 protein family, leading to regulation of endocytic recycling, an important process during mycobacterial infection [49-51].

IPA was used for identification of significantly over-represented GO categories and canonical pathways. Notably, these analyses demonstrated that the processes of cell death, apoptosis and cell survival were over-represented as GO categories and within canonical pathways levels. These pathways included: *Death receptor signalling*; *Apoptosis signalling*; *TNFR2 signalling*; *TNFR1 signalling*; *PTEN signalling*; and *TWEAK signalling*. Inspection of these pathways revealed that the genes encoding TNF-α and the tumour necrosis factor (ligand) superfamily, member 10 (TNFSF-10) protein are highly upregulated. These proteins can, via their respective receptors—tumour necrosis factor receptor superfamily member 1A (TNFRSF-1A) and TNFRSF-10A/TNFRSF-10B—trigger an activation cascade through Fas (TNFRSF6)-associated via death domain (FADD) and TNFRSF1A-associated via death domain (TRADD) [both genes encoding these proteins were upregulated in the present study]. This cascade concludes with the activation of several caspases that are associated with the induction of apoptosis (upregulation of *CASP7* and *CASP8* was observed here), further supporting the role of apoptosis in the host response to mycobacterial infection [25].

IPA analysis also demonstrated the importance of gene products involved with immune cell communication and chemotaxis of immune cells, including the canonical pathways *Communication between innate and adaptive immune cells* and *The role of cytokines in mediating communication between immune cells*. For example, it is well established that macrophage recognition of bacterial pathogen-association molecular patterns (PAMPs) via specific Toll-like receptors (TLRs) [upregulation of *TLR3*; *TLR5*; and *TLR10* and downregulation of *TLR9* was observed here] leads to increased expression of a wide range of NF-κB-inducible cytokines and chemokines. In this regard, *CCL4*, *CCL5*, *CXCL10* and *IL8* were upregulated in the present study. Indeed, the overall pattern of immune gene expression changes observed here using RNA-seq data was consistent with previous work from our group using microarray technology [20].

### Antisense strand transcript expression

The present study identified 0.4 million sequence reads that mapped to the antisense strand and it is important to note that the phenomenon of natural antisense transcription has been observed previously in mammals [52,53]. We identified 6,842 putative natural antisense transcripts (NATs) that were suitable for differential expression analysis of the *M. bovis*-infected MDM relative to the non-infected control MDM. This represents 26.7% of the 25,669 currently annotated *B. taurus* genes, which corresponds to results obtained from previous studies of antisense strand transcription on different mammals including cattle [54-58]. Of the 6,842 NATs detected, 757 were significantly differentially expressed; these comprised 558 that were upregulated and 199 that were downregulated. NATs have been shown to have varying regulatory roles in eukaryotic cells, including transcriptional and post-transcriptional control, splicing event regulation, allele-specific transcript expression, RNA editing and RNA translocation [59-64]. Further analyses identified 694 genes that were differentially expressed from both the sense and antisense strand.

IPA analysis of these 757 antisense strand transcripts revealed enrichment of genes involved in several immune processes including the inflammatory response, pattern recognition receptor signalling, cell death and apoptosis, immune cell movement and interaction, and antigen presentation. These findings suggest that NATs may play a significant role in regulating the host macrophage response following infection with pathogenic agents, including mycobacteria [52,65].

### Comparison with microarray and real time qRT-PCR

The MDM samples infected with *M. bovis* used for the present study had previously been analysed using the Affymetrix® GeneChip® Bovine Genome Array. Consequently, it was possible to directly compare gene expression results from microarray and sense strand RNA-seq data. The percentage of overlapping genes between the two platforms was estimated at 47.8% for the microarray and 37.3% for the RNA-seq data. Similar estimates of correspondence between RNA-seq and microarrays platforms have been reported in studies of the rat and *Candida parapsilosis* transcriptomes [28,66]. However, it should be noted that compared to the study of Su *et al.*[28] we observed a greater correlation between the fold-change in expression for: (1) all common genes that passed filtering detected by both platforms (6,183 genes; Spearman correlation coefficient of 0.858; *P* ≤ 0.001), and (2) the differentially expressed genes common to both platforms (965 genes; Spearman correlation coefficient of 0.935; *P* ≤ 0.001).

There are a number of possible reasons for the differences observed between the two gene expression platforms used in the current study. Firstly, there are differences in the dynamic range of the two platforms: our results show that RNA-seq has a larger dynamic range and sensitivity than the microarray; similar results have recently been obtained by Chen and colleagues [27]. Secondly, the stringent expression thresholds that we have applied to the RNA-seq data in order to reduce the number of false-positive differentially expressed genes, have also reduced the number of genes common to both platforms. Thirdly, the probes on the Affymetrix® GeneChip® Bovine Genome Array are based on sequences from the 3^′^ end of genes and are therefore 3^′^ biased [67]; RNA-seq reads are more randomly distributed across gene transcripts. Fourthly, microarrays are susceptible to cross-hybridisation, particularly among members for the same gene family that can result in elevated false-positive rates [30-32]. Fifthly, there are differences in the statistical models used to detect differentially expressed genes for the two platforms (for example, analysis of the microarray data involve moderated *t*-tests; analysis of the RNA-seq data using edgeR involved a negative binomial distribution) [28,68-71]. Sixthly, differences in the bovine genome resources used to design the Affymetrix® GeneChip® Bovine Genome Array (May 2005, Gene Expression Omnibus platform accession number GPL2112) and the *B. taurus* reference genome (Btau 4.0.63 genome release) used to analyse the RNA-seq data may also impact on the analysis as recently proposed in a study of the yeast transcriptome [72]. Seventhly, it has been demonstrated that increased sequencing depth also contributes to greater correspondence between RNA-seq and microarray platforms, such as that recently observed in a study of the *Saccharomyces cerevisiae* transcriptome [36,72].

It is important to note, however, that despite the moderate concordance in the number of differentially expressed genes common to both transcriptomic platforms, we did observe a high concordance between the GO categories and canonical pathways identified by IPA analysis. This indicates that the RNA-seq and microarray platforms both provide gene expression data that can be used for meaningful biological interpretation.

## Conclusions

The results from the present study highlight the ability of the RNA-seq technologies to reveal novel features of the bovine macrophage transcriptome in response to infection with *M. bovis*, including the detection of putative NAT expression. These transcriptional signatures highlight the complex interactions between host macrophages and the mycobacterial pathogen during infection. Further analyses involving the comparison of RNA-seq-generated gene expression profiles in bovine macrophages to non-pathogenic mycobacteria, such as *M. bovis*-BCG, may enable fine-scaled interrogation of key cellular pathways that contribute to the development of pathology.

## Methods

### MDM preparation, MDM infection and RNA purification

The materials and methods used to isolate, purify and infect bovine MDM with *M. bovis* have been previously described in detail by us [20]. Briefly, MDM were purified from peripheral blood mononuclear cells prepared from whole blood extracted from seven age-matched (four-years old) Holstein-Friesian females (Additional file 1: Table S1). All seven animals were selected from a herd without a recent history of bovine tuberculosis infection, which was confirmed using the single intradermal tuberculin test. MDM were cultured over an eight-day period with routine changing of culture media every two days. MDM were visually inspected using microscopy, counted and seeded in 24-well tissue culture plates at a density of 2 × 10^5^ cells per well prior to infection.

For the MDM infections, 4 × 10^5^ *M. bovis* cells (as determined from bacterial cell counts performed using a Petroff Hausser chamber and confirmed by colony-forming unit [cfu] counts) were added to each tissue culture plate well (MOI 2:1). The *M. bovis* M2137 strain bearing the SB0142 spoligotype was used for the infection experiments and non-infected control MDM received culture media only. Both non-infected control and infected MDM were prepared in adjacent duplicate tissue culture plate wells. The culture media in all wells for both non-infected control and infected MDM was replaced 2 h post-infection with fresh culture media and plates were then re-incubated at 37°C, 5% CO_2_ until the MDM were harvested. Culturing of *M. bovis* and the MDM infections were performed in a Biosafety Containment Level 3 (CL3) laboratory.

Infected and non-infected control MDM were harvested using RLT buffer from the RNeasy Mini kit (Qiagen Ltd., Crawley, UK) supplemented with 1% β-mercaptoethanol (Sigma-Aldrich Ireland Ltd., Dublin, Ireland) 24 h post-infection. For each control and treatment, MDM lysates from duplicate culture plate wells were pooled and stored at -80°C until required for RNA extraction. All RNA extractions were performed in the CL3 laboratory using an RNeasy kit incorporating an on-column DNase treatment step according to the manufacturer’s instructions (Qiagen). RNA quantity and quality was ascertained using a NanoDrop™ 1000 spectrophotometer (Thermo Fisher Scientific Ltd., Waltham, MA, USA) and an Agilent 2100 Bioanalyzer with the RNA 6000 Nano LabChip kit (Agilent Technologies Ltd., Cork, Ireland). All samples displayed a 260/280 ratio greater than 2.0 and RNA integrity numbers ≥ 8.5 [73].

### Strand-specific RNA-seq library preparation

In total, 14 strand-specific RNA libraries for high-throughput sequencing were prepared (seven libraries for each treatment: *M. bovis*-infected and control samples) using 200 ng of total RNA. Total RNA was first heated at 65°C for 5 min to disrupt any secondary structure. Purification of poly(A) RNA was performed using a Dynabeads® mRNA DIRECT™ Micro Kit according to the manufacturer’s instructions (Invitrogen™/Life Technologies Ltd., Paisley, UK). Purified poly(A) RNA was then fragmented using 1× RNA Fragmentation Reagent (Ambion®/Life Technologies Corporation, Warrington, UK) for 5 min at 70°C and precipitated using 68 mM sodium acetate pH 5.2 (Ambion), 227 ng/μl glycogen (Ambion) and 30 μl of 100% ethanol (Sigma-Aldrich Ltd., Dublin, Ireland). Pellets were washed with 80% ethanol, air-dried for 10 min at room temperature and re-suspended in 10.5 μl DNase- and RNase-free water.

Synthesis of first strand cDNA was performed by incubating fragmented RNA with 261 mM Random Hexamer Primers (Invitrogen), 1× first strand buffer (Invitrogen); 10 mM DTT (Invitrogen); 0.5 mM dNTPs; 20 U RNaseOUT™ Recombinant Ribonuclease Inhibitor; and 200 U SuperScript® II Reverse Transcriptase (Invitrogen) at 25°C for 10 min, at 42°C for 50 min, and 70°C for 15 min. First strand synthesis reaction mixtures were purified using MicroSpin™ G-50 columns according to the manufacturer’s instructions (GE Healthcare UK Ltd., Little Chalfont, Buckinghamshire, UK).

Second strand cDNA synthesis, involving the incorporation of uracil, was performed by adding the first strand cDNA synthesis reaction to a second strand reaction mix consisting of 0.065× first strand buffer (Invitrogen); 1× second strand buffer (Invitrogen); a dNTP mix consisting of a final concentration of 0.3 mM dATP, dCTP, dGTP (Sigma-Aldrich) and 0.3 mM dUTP (Bioline Reagents Ltd., London, UK); 1 mM DTT (Invitrogen); 2 U RNase H (Invitrogen) and 50 U *E. coli* DNA Polymerase I (Invitrogen). Reactions were incubated at 16°C for 2.5 h. The double stranded cDNA was subsequently purified using a QIAquick PCR Purification kit (Qiagen) according to the manufacturer’s instructions and eluted in 30 μl of the provided elution buffer.

Blunt-end repair of cDNA was performed in a 100 μl reaction containing 1× T4 DNA ligase buffer with 10 mM dATP (New England Biolabs® Inc., MA, USA), 0.4 mM of each dNTP (Invitrogen), 15 U T4 DNA polymerase (New England Biolabs), 5 U DNA Polymerase I Large [Klenow] Fragment (New England Biolabs) and 50 U T4 polynucleotide kinase (New England Biolabs). Reactions were incubated at 20°C for 30 min and the cDNA was then purified using a QIAquick PCR Purification Kit (Qiagen) according to the manufacturer’s instructions and eluted in 32 μl of the provided elution buffer.

To facilitate Illumina® GA adaptor ligation, a single ‘A’ base was added to the 3^′^ ends of the blunt-end repaired cDNA samples. 32 μl of purified phosphorylated blunt end-repaired cDNA was included in a final 50 μl reaction mixture containing: 1× Klenow fragment buffer (New England Biolabs); 0.2 mM dATP (Invitrogen), and 15 U Klenow fragment with 3^′^-to-5^′^ exonuclease activity (New England Biolabs). Reactions were incubated at 37°C for 30 min, after which cDNA was purified using a QIAquick MinElute Kit (Qiagen) according to the manufacturer’s instructions and eluted in 21 μl of the provided elution buffer.

Illumina® RNA-seq adaptor ligation reactions (50 μl volumes) involved incubation of 21 μl of phosphorylated blunt-ended cDNA containing a 3^′^-dATP overhang with 1× Quick DNA ligase buffer (New England Biolabs); 30 nM custom indexed single-read adaptors [Additional file 1: Table S1] and 15 U T4 DNA ligase (Invitrogen). Reaction mixes were incubated at room temperature for 15 min and purified using a QIAquick MinElute Kit according to the manufacturer’s instructions (Qiagen) and eluted in 10 μl of the provided elution buffer. Adaptor-ligated cDNA was gel-purified using 2.5% agarose gels stained with 1 μg/ml ethidium bromide (Invitrogen). Gels were electrophoresed at 100 Volts using 1× TAE buffer (Invitrogen) for 75 min at room temperature. Size fractionated bands corresponding to 200 bp (+50 bp) were excised from each sample and purified using a QIAquick Gel Extraction kit (Qiagen) according to the manufacturer’s instructions and eluted in 30 μl of elution buffer.

To generate strand-specific RNA-seq libraries, the second strand of the gel-purified adapter-ligated cDNA containing uracil was digested enzymatically in 30 μl reaction volumes containing 1× Uracil-DNA Glycosylase buffer and 1 U Uracil-DNA Glycosylase (Bioline). Reactions were incubated at 37°C for 15 min followed by 94°C for 10 min.

PCR enrichment amplifications (50 μl) containing 15 μl of second strand-digested, adaptor-ligated cDNA; 1× Phusion® High-Fidelity DNA polymerase buffer (New England Biolabs); 334 nM each Illumina® PCR primer (Illumina® Inc., San Diego, CA, USA); 0.4 mM each of dATP, dCTP, DGTP and dTTP (Invitrogen) and 1 U Phusion® High-Fidelity DNA polymerase (New England Biolabs). PCR amplification reactions consisted of an initial denaturation step of 98°C for 30 seconds, 18 cycles of 98°C for 10 seconds, 65°C for 30 seconds and 72°C for 30 seconds, followed by a final extension step of 72°C for 5 min. PCR products were visualised following electrophoresis on a 2% agarose gel stained with ethidium bromide (0.6 μg/ml; Invitrogen) and purified to remove PCR-generated adaptor-dimers using an Agencourt AMPure XP kit (Beckman Coulter Genomics, Danvers, MA, USA) according to the manufacturer’s instructions with final elution in 30 μl of 1× TE buffer.

All RNA-seq libraries were quantified using a Qubit® Fluorometer and Qubit® double stranded DNA High Sensitivity Assay Kit (Invitrogen). RNA-seq library quality was assessed using an Agilent Bioanalyzer and Agilent High sensitivity DNA chip (Agilent) and confirmed that library insert sizes were ~200-250 bp for all individual libraries. Individual RNA-seq libraries were standardised and pooled in equimolar quantities (10 μM for each individual library). The quantity and quality of the final pooled library was assessed as described above prior to sequencing.

Cluster generation and sequencing of the pooled RNA-seq library was carried out on an Illumina® Cluster Station and Illumina® Genome Analyzer IIx sequencer according to the manufacturer’s instructions (Illumina). The pooled library was sequenced as single-end read 84-mers using Illumina® version 4.0 sequencing kits and the standard Illumina® Genome Analyzer II_x_ pipeline. The Illumina® Sequencing Control Software version 2.9 and Real Time Analysis version 1.9 software packages were used for real-time tracking of the sequencing run, real-time image processing, the generation of base intensity values and base calling. These RNA-seq data have been deposited in the NCBI Gene Expression Omnibus (GEO) database with experiment series accession number GSE45439.

### Statistical analysis of RNA-seq data

Sequence reads obtained from seven lanes of the Illumina flow cell were deconvoluted into 14 individual libraries using the unique indexed barcoded adapters. A Perl script was used to screen adapter artefacts, removing any reads containing a full-length match to the 33 nucleotide Illumina® adapter sequence, allowing up to four mismatches. Read quality was then assessed using FastQC software [version 0.9] (http://www.bioinformatics.bbsrc.ac.uk/projects/fastqc), revealing low Phred scores at the 3^′^ end of sequence reads. Consequently, nine nucleotides were trimmed from the 3^′^ ends of all sequence reads using the FASTX Toolkit [version 0.0.13] (http://hannonlab.cshl.edu/fastx_toolkit) generating usable reads of 69 nucleotides.

Deconvoluted quality-checked sequence reads were aligned to the *B. taurus* reference genome (Btau 4.0.63 genome release) with the TopHat splice junction mapper [version 1.3.0] [74,75], which aligns reads using the Bowtie aligner (version 0.12.7). The Bowtie alignment procedure was configured for strand-specific libraries and to filter only unique hits to the reference genome sequence. In addition, all sequence reads were aligned to the *M. bovis* AF2122/97 chromosome, complete genome (accession number NC_002945.3) as detailed above. To obtain raw counts per transcript, HTSeq package [version 0.5.3p1] (http://www-huber.embl.de/users/anders/HTSeq/doc/overview.html) was used on alignment files in BAM format. HTSeq-count was used with option overlap resolution mode set to intersection non-empty. Counts of uniquely-mapped reads were obtained for all bovine Ensembl genes and transcripts, with separate counts obtained for sense and antisense DNA strands.

Once raw counts were obtained from HTseq-count, analysis of differential expression was performed in the R statistical programming environment [76] using the edgeR (version 2.2.5) Bioconductor package [70,77] and lattice [version 0.19-30] (http://lattice.r-forge.r-project.org). First, quality checks were performed by plotting the density of counts per feature (a feature being a gene or transcript generated respectively from the sense or antisense strand) for each sample and also by generating a multidimensional scaling plot of the RNA-seq data. The edgeR software package was used to determine differential expression using the paired-sample statistical test. Filtering of lowly expressed features was performed by retaining only features with at least one count per million in three or more libraries. A normalisation factor was calculated using the default trimmed mean of M values (TMM) method [70,77], and the dispersion parameter for each feature was estimated as the Cox-Reid common dispersion method in the edgeR package. Differential expression was evaluated by fitting a negative binomial generalized linear model for each feature and then adjusting the *P*-value for multiple testing using the Benjamini-Hochberg correction [78] with a false discovery rate (FDR) of 0.05.

For RNA-seq dynamic range analysis we used two different normalisation strategies: (1) RPKM values generated by the edgeR package; and (2) quantile normalisation of RNA-seq data performed using the Linear Models for Microarray Data (LIMMA) package [71]. RPKM and quantile-normalised counts were not used for differential expression analysis.

### Reanalysis and reannotation of Affymetrix® GeneChip® Bovine Genome Array data

The RNA samples analysed in the current study were previously analysed by us using the Affymetrix® GeneChip® Bovine Genome Array [20]. These microarray data have been deposited in the NCBI GEO database with experiment series accession number GSE33309. In order to compare gene expression profiles from these samples using RNA-seq and microarray technologies, we first reanalysed the microarray data using a series of Bioconductor packages.

Firstly, quality control analysis was performed on all microarrays using the Simpleaffy software package [79]. Then the raw microarray expression values were normalised using the gcRMA package [80]. The raw data followed an additional normalisation step using the Factor Analysis for Robust Microarray Summarization (FARMS) algorithm to remove probe sets with high noise:signal ratios [81], these normalised data were then further subjected to filtering for informative probe sets using informative/non-informative calls (I/NI-calls) package [82]. The obtained I/NI filtering list was then applied to the gcRMA normalised data to remove all non-informative probes. Differentially expressed genes were identified from the filtered gcRMA normalised data using the LIMMA package. The Benjamini-Hochberg multiple testing correction method [78] was applied to all differentially expressed genes to minimise the FDR and adjusted *P*-values for differentially expressed genes were calculated. Probe sets displaying differential expression between control and infected samples were annotated to Ensembl gene IDs using biomaRt package [83].

Supplementary statistical analyses were performed using the SPSS software package (version 20; http://www-01.ibm.com/software/analytics/spss).

### IPA analyses

Ingenuity® Systems Pathway Analysis (IPA, Ingenuity Systems, Redwood City, CA, USA; release date November 2012) was used to identify canonical pathways and functional processes of biological importance within the list of differentially expressed genes identified with RNA-seq and microarray platforms. The Ingenuity® Knowledge Base contains the largest database of manually-curated and experimentally-validated physical, transcriptional and enzymatic molecular interactions. Furthermore, each interaction in the Ingenuity® Knowledge Base is supported by previously published information.

Functional analysis of genes was performed using IPA to characterise the GO categories of differentially expressed genes between the control and *M. bovis*-infected MDM. For this, IPA performed an over-representation analysis that categorises differentially expressed genes into functional groups using the Ingenuity® Knowledge Base. Each category in IPA is ranked based on the number of differentially expressed genes in each functional group. The right-tailed Fisher’s exact test was used to calculate a *P*-value for each GO category assigned to differentially expressed genes.

Ingenuity® Systems Pathway Analysis contains a large library of known canonical pathways that were overlaid with the differentially expressed genes to identify major biological pathways associated with *M. bovis* infection in MDM. The significance of the association between differentially expressed genes and each canonical pathway was assessed using two methods. Firstly, a ratio was estimated from the number of molecules from the differentially expressed gene data set that map to each pathway, compared to the total number of molecules that map to the canonical pathway based on the reference gene list; and secondly, a Fisher’s exact test that generates a *P*-value for the assignment of the differentially expressed genes to a particular canonical pathway compared to the reference gene list. Canonical pathways were then overlaid with the expression values of the differentially expressed genes.

## Competing interests

The authors declare that they have no competing interests.

## Authors’ contributions

Conceived and designed the experiments: NCN, DAM, MT, JAB, AJL, BJL, EG, SVG, DEM. Performed the experiments: NCN, DAM, MT, JAB, KMC. Analysed the data: NCN, SDEP, DAM, MT, KRA, KEK, KH, JAB. Prepared and edited the manuscript: NCN, DAM, SDEP, MT, KRA, KEK, KH, AJL, BJL, EG, SVG, DEM. All authors read and approved the manuscript.

## Supplementary Material

Additional file 1: Table S1 RNA-seq libraries information. The indexed adapter sequence, animal sample ID, sample treatment, pooling strategy, and sequencing read information (number and percentage) before and after data filtering are given for each RNA-seq library. Click here for file

Additional file 2: Figure S1 Density plot of the distribution of reads per gene. Density plots of the number of sequence reads (in log_10_ space) per gene for each RNA-seq library sample. Click here for file

Additional file 3: Table S2 The list of all significant differentially expressed genes detected following *M. bovis* infection based on RNA-seq sense strand data. For each differentially expressed gene is shown its gene name, log_2_ fold-change, *P*-value, adjusted *P*-value (Benjamini-Hochberg correction), description and Ensembl gene ID. The biomaRt package and the *B. taurus* reference genome were used to obtain gene names and gene descriptions. Genes without names or descriptions are stated here as “not available”. Click here for file

Additional file 4: Table S3 GO categories identified using IPA based on RNA-seq sense strand data. The top ranking GO categories identified by IPA based on RNA-seq sense strand data are ranked according to *P*-values. Click here for file

Additional file 5: Table S4 Significant canonical pathways identified using IPA based on RNA-seq sense strand data. The canonical pathways identified by IPA based on RNA-seq sense strand data are ranked according to *P*-values. The ratio indicates the number of differentially expressed genes involved in each canonical pathway divided by the total number of genes/molecules within each pathway according to the IPA Knowledge Base. Click here for file

Additional file 6: Table S5 The list of all significant differentially expressed genes detected following *M. bovis* infection based on RNA-seq antisense strand data. For each differentially expressed gene is shown its gene name, log_2_ fold-change, *P*-value, adjusted *P*-value (Benjamini-Hochberg correction), description and Ensembl gene ID. The biomaRt package and the *B. taurus* reference genome were used to obtain gene names and gene descriptions. Genes without names or descriptions are stated here as “not available”. Click here for file

Additional file 7: Figure S2 Integrative Genomics Viewer (IGV) screen capture of reads mapping to *SPTB* gene. This figure shows the distribution of sense (represented in red) and antisense (represented in blue) strand reads that mapped to the 3′ end of spectrin, beta, erythrocytic gene (*SPTB*). Click here for file

Additional file 8: Table S6 GO categories identified using IPA based on RNA-seq antisense strand data. The top ranking GO categories identified by IPA based on RNA-seq antisense strand data are ranked according to *P*-values. Click here for file

Additional file 9: Table S7 Significant canonical pathways identified using IPA based on RNA-seq antisense strand data. The canonical pathways identified by IPA based on RNA-seq antisense strand data are ranked according to *P*-values. The ratio indicates the number of differentially expressed genes involved in each canonical pathway divided by the total number of genes/molecules within each pathway according to the IPA® Knowledge Base. Click here for file

Additional file 10: Figure S3 Multi-dimensional scale plot of all *M. bovis*-infected and control samples based on Microarray data. Dimension 1 and dimension 2 separate all 12 samples based on the expression value of the 11,790 probes (based on microarray data only) that passed all data filtering criteria prior to differential gene expression analysis. Click here for file

Additional file 11: Table S8 The list of all significant differentially expressed genes detected following *M. bovis* infection based on microarray data. For each differentially expressed gene is shown its gene name, log_2_ fold-change, *P*-value, adjusted *P*-value (Benjamini-Hochberg correction), description and Ensembl gene ID. The biomaRt package and the *B. taurus* reference genome were used to obtain gene names and gene descriptions. Genes without names or descriptions are stated here as “not available”. Click here for file

Additional file 12: Table S9 List of all significant differentially expressed genes detected following *M. bovis* infection common to both microarray and RNA-seq sense strand data. For each differentially expressed gene is shown its Ensembl gene ID, gene name, description, log_2_ fold-change, *P*-value and adjusted *P*-value (Benjamini-Hochberg correction) based on microarray and RNA-seq sense strand data. The biomaRt package and the *B. taurus* reference genome were used to obtain gene names and gene descriptions. Genes without names or descriptions are stated here as “not available”. Click here for file

Additional file 13: Figure S4 Density plots of log_2_ mean CPM and log_2_ mean hybridisation intensities for differentially expressed genes unique and common to both platforms. This analysis was performed for each platform/treatment group. DEG, differentially expressed genes. Click here for file

Additional file 14: Table S10 GO categories identified using IPA based on microarray data. The top ranking GO categories identified by IPA based on microarray data are ranked according to *P*-values. Click here for file

Additional file 15: Table S11 GO categories identified using IPA based on differentially expressed genes common to both microarray and RNA-seq sense strand data. The top ranking GO categories identified by IPA based on differentially expressed genes common to both microarray and RNA-seq sense strand data are ranked according to *P*-values. Click here for file

Additional file 16: Table S12 Significant canonical pathways identified using IPA based on microarray data. The canonical pathways identified by IPA based on microarray data are ranked according to *P*-values. The ratio indicates the number of differentially expressed genes involved in each canonical pathway divided by the total number of genes/molecules within each pathway according to the IPA® Knowledge Base. Click here for file

Additional file 17: Table S13 Significant canonical pathways identified using IPA based on differentially expressed genes common to both microarray and RNA-seq sense strand data. The canonical pathways identified by IPA based on differentially expressed genes common to both microarray and RNA-seq sense strand data are ranked according to *P*-values. The ratio indicates the number of differentially expressed genes involved in each canonical pathway divided by the total number of genes/molecules within each pathway according to the IPA® Knowledge Base. Click here for file

## References

[B1] Brosch R Gordon SV Marmiesse M Brodin P Buchrieser C Eiglmeier K Garnier T Gutierrez C Hewinson G Kremer K A new evolutionary scenario for the *Mycobacterium tuberculosis* complex Proc Natl Acad Sci USA 2002 99 6 3684 3689 10.1073/pnas.052548299 11891304 PMC122584

[B2] Djelouadji Z Raoult D Drancourt M Palaeogenomics of *Mycobacterium tuberculosis*: epidemic bursts with a degrading genome Lancet Infect Dis 2011 11 8 641 650 10.1016/S1473-3099(11)70093-7 21672667

[B3] Wirth T Hildebrand F Allix-Beguec C Wolbeling F Kubica T Kremer K van Soolingen D Rusch-Gerdes S Locht C Brisse S Origin, spread and demography of the *Mycobacterium tuberculosis* complex PLoS Pathog 2008 4 9 e1000160 10.1371/journal.ppat.1000160 18802459 PMC2528947

[B4] Smith NH Gordon SV de la Rua-Domenech R Clifton-Hadley RS Hewinson RG Bottlenecks and broomsticks: the molecular evolution of *Mycobacterium bovis* Nat Rev Microbiol 2006 4 9 670 681 10.1038/nrmicro1472 16912712

[B5] Good M Bovine tuberculosis eradication in Ireland Irish Veterinary Journal 2006 59 3 154 162

[B6] More SJ Good M The tuberculosis eradication programme in Ireland: a review of scientific and policy advances since 1988 Vet Microbiol 2006 112 2–4 239 251 16337345 10.1016/j.vetmic.2005.11.022

[B7] Pollock JM Neill SD *Mycobacterium bovis* infection and tuberculosis in cattle Vet J 2002 163 2 115 127 10.1053/tvjl.2001.0655 12093187

[B8] Harding CV Boom WH Regulation of antigen presentation by *Mycobacterium tuberculosis*: a role for Toll-like receptors Nat Rev Microbiol 2010 8 4 296 307 10.1038/nrmicro2321 20234378 PMC3037727

[B9] Kunnath-Velayudhan S Gennaro ML Immunodiagnosis of tuberculosis: a dynamic view of biomarker discovery Clin Microbiol Rev 2011 24 4 792 805 10.1128/CMR.00014-11 21976609 PMC3194832

[B10] Pollock JM Welsh MD McNair J Immune responses in bovine tuberculosis: towards new strategies for the diagnosis and control of disease Vet Immunol Immunopathol 2005 108 1–2 37 43 16150494 10.1016/j.vetimm.2005.08.012

[B11] Waters WR Palmer MV Thacker TC Davis WC Sreevatsan S Coussens P Meade KG Hope JC Estes DM Tuberculosis immunity: opportunities from studies with cattle Clin Dev Immunol 2011 2011 768542 21197095 10.1155/2011/768542 PMC3004413

[B12] Sundaramurthy V Pieters J Interactions of pathogenic mycobacteria with host macrophages Microbes Infect 2007 9 14–15 1671 1679 18023233 10.1016/j.micinf.2007.09.007

[B13] Meena LS Rajni Survival mechanisms of pathogenic *Mycobacterium tuberculosis* H37Rv FEBS J 2010 277 11 2416 2427 10.1111/j.1742-4658.2010.07666.x 20553485

[B14] Walzl G Ronacher K Hanekom W Scriba TJ Zumla A Immunological biomarkers of tuberculosis Nat Rev Immunol 2011 11 5 343 354 10.1038/nri2960 21475309

[B15] MacHugh DE Gormley E Park SD Browne JA Taraktsoglou M O’Farrelly C Meade KG Gene expression profiling of the host response to *Mycobacterium bovis* infection in cattle Transbound Emerg Dis 2009 56 6–7 204 214 19486308 10.1111/j.1865-1682.2009.01082.x

[B16] Widdison S Watson M Coffey TJ Early response of bovine alveolar macrophages to infection with live and heat-killed *Mycobacterium bovis* Dev Comp Immunol 2011 35 5 580 591 10.1016/j.dci.2011.01.001 21232552

[B17] Meade KG Gormley E Doyle MB Fitzsimons T O’Farrelly C Costello E Keane J Zhao Y MacHugh DE Innate gene repression associated with *Mycobacterium bovis* infection in cattle: toward a gene signature of disease BMC Genomics 2007 8 400 10.1186/1471-2164-8-400 17974019 PMC2213678

[B18] Kabara E Kloss CC Wilson M Tempelman RJ Sreevatsan S Janagama H Coussens PM A large-scale study of differential gene expression in monocyte-derived macrophages infected with several strains of *Mycobacterium avium* subspecies *paratuberculosis* Brief Funct Genomics 2010 9 3 220 237 10.1093/bfgp/elq009 20495212

[B19] MacHugh D Taraktsoglou M Killick K Nalpas N Browne J Park S Hokamp K Gormley E Magee D Pan-genomic analysis of bovine monocyte-derived macrophage gene expression in response to *in vitro* infection with *Mycobacterium avium* subspecies *paratuberculosis* Vet Res 2012 43 1 25 10.1186/1297-9716-43-25 22455317 PMC3411445

[B20] Magee DA Taraktsoglou M Killick KE Nalpas NC Browne JA Park SDE Conlon KM Lynn DJ Hokamp K Gordon SV Global gene expression and systems biology analysis of bovine monocyte-derived macrophages in response to *in vitro* challenge with *Mycobacterium bovis* PLoS One 2012 7 2 e32034 10.1371/journal.pone.0032034 22384131 PMC3284544

[B21] Killick KE Browne JA Park SD Magee DA Martin I Meade KG Gordon SV Gormley E O’Farrelly C Hokamp K Genome-wide transcriptional profiling of peripheral blood leukocytes from cattle infected with *Mycobacterium bovis* reveals suppression of host immune genes BMC Genomics 2011 12 1 611 10.1186/1471-2164-12-611 22182502 PMC3292584

[B22] Wilhelm BT Landry JR RNA-Seq-quantitative measurement of expression through massively parallel RNA-sequencing Methods 2009 48 3 249 257 10.1016/j.ymeth.2009.03.016 19336255

[B23] Ozsolak F Milos PM RNA sequencing: advances, challenges and opportunities Nat Rev Genet 2011 12 2 87 98 10.1038/nrg2934 21191423 PMC3031867

[B24] Roy NC Altermann E Park ZA McNabb WC A comparison of analog and next-generation transcriptomic tools for mammalian studies Brief Funct Genomics 2011 10 3 135 150 10.1093/bfgp/elr005 21389008

[B25] Behar SM Martin CJ Booty MG Nishimura T Zhao X Gan HX Divangahi M Remold HG Apoptosis is an innate defense function of macrophages against *Mycobacterium tuberculosis* Mucosal Immunol 2011 4 3 279 287 10.1038/mi.2011.3 21307848 PMC3155700

[B26] GeneChip® Bovine Genome Array data sheethttp://media.affymetrix.com/support/technical/datasheets/bovine_datasheet.pdf23613893

[B27] Chen H Liu Z Gong S Wu X Taylor WL Williams RW Matta SG Sharp BM Genome-wide gene expression profiling of nucleus accumbens neurons projecting to ventral pallidum using both microarray and transcriptome sequencing Frontiers in neuroscience 2011 5 98 21886604 10.3389/fnins.2011.00098 PMC3155868

[B28] Su Z Li Z Chen T Li QZ Fang H Ding D Ge W Ning B Hong H Perkins RG Comparing next-generation sequencing and microarray technologies in a toxicological study of the effects of aristolochic acid on rat kidneys Chem Res Toxicol 2011 24 9 1486 1493 10.1021/tx200103b 21834575

[B29] Lesho E Forestiero FJ Hirata MH Hirata RD Cecon L Melo FF Paik SH Murata Y Ferguson EW Wang Z Transcriptional responses of host peripheral blood cells to tuberculosis infection Tuberculosis (Edinb) 2011 91 5 390 399 10.1016/j.tube.2011.07.002 21835698

[B30] Bradford JR Hey Y Yates T Li Y Pepper SD Miller CJ A comparison of massively parallel nucleotide sequencing with oligonucleotide microarrays for global transcription profiling BMC Genomics 2010 11 282 10.1186/1471-2164-11-282 20444259 PMC2877694

[B31] Liu S Lin L Jiang P Wang D Xing Y A comparison of RNA-Seq and high-density exon array for detecting differential gene expression between closely related species Nucleic Acids Res 2011 39 2 578 588 10.1093/nar/gkq817 20864445 PMC3025565

[B32] Marioni JC Mason CE Mane SM Stephens M Gilad Y RNA-seq: an assessment of technical reproducibility and comparison with gene expression arrays Genome Res 2008 18 9 1509 1517 10.1101/gr.079558.108 18550803 PMC2527709

[B33] Grabherr MG Haas BJ Yassour M Levin JZ Thompson DA Amit I Adiconis X Fan L Raychowdhury R Zeng Q Full-length transcriptome assembly from RNA-Seq data without a reference genome Nat Biotech 2011 29 7 644 652 10.1038/nbt.1883 PMC3571712 21572440

[B34] Garber M Grabherr MG Guttman M Trapnell C Computational methods for transcriptome annotation and quantification using RNA-seq Nat Meth 2011 8 6 469 477 10.1038/nmeth.1613 21623353

[B35] Toung JM Morley M Li M Cheung VG RNA-sequence analysis of human B-cells Genome Res 2011 21 6 991 998 10.1101/gr.116335.110 21536721 PMC3106332

[B36] Tarazona S Garcia-Alcalde F Dopazo J Ferrer A Conesa A Differential expression in RNA-seq: a matter of depth Genome Res 2011 21 12 2213 2223 10.1101/gr.124321.111 21903743 PMC3227109

[B37] Wang Y Ghaffari N Johnson CD Braga-Neto UM Wang H Chen R Zhou H Evaluation of the coverage and depth of transcriptome by RNA-Seq in chickens BMC Bioinformatics 2011 12 Suppl 10 S5 10.1186/1471-2105-12-S10-S5 22165852 PMC3236848

[B38] Blanco FC Soria M Bianco MV Bigi F Transcriptional response of peripheral blood mononuclear cells from cattle infected with *Mycobacterium bovis* PLoS One 2012 7 7 e41066 10.1371/journal.pone.0041066 22815916 PMC3397951

[B39] Mirrakhimov AE Kerimkulova AS Lunegova OS Moldokeeva CB Zalesskaya YV Abilova SS Sovhozova NA Aldashev AA Mirrakhimov EM An association between TRP64ARG polymorphism of the B3 adrenoreceptor gene and some metabolic disturbances Cardiovasc Diabetol 2011 10 89 10.1186/1475-2840-10-89 21992420 PMC3215178

[B40] Lirussi F Rakotoniaina Z Madani S Goirand F Breuiller-Fouche M Leroy MJ Sagot P Morrison JJ Dumas M Bardou M ADRB3 adrenergic receptor is a key regulator of human myometrial apoptosis and inflammation during chorioamnionitis Biol Reprod 2008 78 3 497 505 10.1095/biolreprod.107.064444 17989355

[B41] Yu JJ Gaffen SL Interleukin-17: a novel inflammatory cytokine that bridges innate and adaptive immunity Front Biosci 2008 13 170 177 10.2741/2667 17981535

[B42] Molenaar AJ Harris DP Rajan GH Pearson ML Callaghan MR Sommer L Farr VC Oden KE Miles MC Petrova RS The acute-phase protein serum amyloid A3 is expressed in the bovine mammary gland and plays a role in host defence Biomarkers 2009 14 1 26 37 10.1080/13547500902730714 19283521

[B43] Bettler B Kaupmann K Mosbacher J Gassmann M Molecular structure and physiological functions of GABA(B) receptors Physiol Rev 2004 84 3 835 867 10.1152/physrev.00036.2003 15269338

[B44] Chapman RW Hey JA Rizzo CA Bolser DC GABAB receptors in the lung Trends Pharmacol Sci 1993 14 1 26 29 10.1016/0165-6147(93)90110-6 8382886

[B45] Dicpinigaitis PV Dobkin JB Rauf K Aldrich TK Inhibition of capsaicin-induced cough by the gamma-aminobutyric acid agonist baclofen J Clin Pharmacol 1998 38 4 364 367 10.1002/j.1552-4604.1998.tb04436.x 9590464

[B46] Halbleib JM Nelson WJ Cadherins in development: cell adhesion, sorting, and tissue morphogenesis Genes Dev 2006 20 23 3199 3214 10.1101/gad.1486806 17158740

[B47] Kadarmideen HN Ali AA Thomson PC Muller B Zinsstag J Polymorphisms of the *SLC11A1* gene and resistance to bovine tuberculosis in African Zebu cattle Anim Genet 2011 42 6 656 658 10.1111/j.1365-2052.2011.02203.x 22035008

[B48] Somia NV Schmitt MJ Vetter DE Van Antwerp D Heinemann SF Verma IM LFG: an anti-apoptotic gene that provides protection from Fas-mediated cell death Proc Natl Acad Sci USA 1999 96 22 12667 12672 10.1073/pnas.96.22.12667 10535980 PMC23041

[B49] Roberts RC Peden AA Buss F Bright NA Latouche M Reilly MM Kendrick-Jones J Luzio JP Mistargeting of SH3TC2 away from the recycling endosome causes Charcot-Marie-Tooth disease type 4C Hum Mol Genet 2010 19 6 1009 1018 10.1093/hmg/ddp565 20028792 PMC2830826

[B50] Halaas O Steigedal M Haug M Awuh JA Ryan L Brech A Sato S Husebye H Cangelosi GA Akira S Intracellular *Mycobacterium avium* intersect transferrin in the Rab11(+) recycling endocytic pathway and avoid lipocalin 2 trafficking to the lysosomal pathway J Infect Dis 2010 201 5 783 792 10.1086/650493 20121435 PMC2862295

[B51] Stendel C Roos A Kleine H Arnaud E Ozcelik M Sidiropoulos PN Zenker J Schupfer F Lehmann U Sobota RM SH3TC2, a protein mutant in Charcot-Marie-Tooth neuropathy, links peripheral nerve myelination to endosomal recycling Brain 2010 133 Pt 8 2462 2474 20826437 10.1093/brain/awq168

[B52] Wang F Hu S Liu W Qiao Z Gao Y Bu Z Deep-sequencing analysis of the mouse transcriptome response to infection with *Brucella melitensis* strains of differing virulence PLoS One 2011 6 12 e28485 10.1371/journal.pone.0028485 22216095 PMC3247208

[B53] ‘t Hoen PA Ariyurek Y Thygesen HH Vreugdenhil E Vossen RH de Menezes RX Boer JM van Ommen GJ den Dunnen JT Deep sequencing-based expression analysis shows major advances in robustness, resolution and inter-lab portability over five microarray platforms Nucleic Acids Res 2008 36 21 e141 10.1093/nar/gkn705 18927111 PMC2588528

[B54] Grigoriadis A Oliver GR Tanney A Kendrick H Smalley MJ Jat P Neville AM Identification of differentially expressed sense and antisense transcript pairs in breast epithelial tissues BMC Genomics 2009 10 324 10.1186/1471-2164-10-324 19615061 PMC2721853

[B55] Yelin R Dahary D Sorek R Levanon EY Goldstein O Shoshan A Diber A Biton S Tamir Y Khosravi R Widespread occurrence of antisense transcription in the human genome Nat Biotechnol 2003 21 4 379 386 10.1038/nbt808 12640466

[B56] Galante PA Vidal DO de Souza JE Camargo AA de Souza SJ Sense-antisense pairs in mammals: functional and evolutionary considerations Genome Biol 2007 8 3 R40 10.1186/gb-2007-8-3-r40 17371592 PMC1868933

[B57] Zhang Y Liu XS Liu QR Wei L Genome-wide *in silico* identification and analysis of *cis* natural antisense transcripts (*cis*-NATs) in ten species Nucleic Acids Res 2006 34 12 3465 3475 10.1093/nar/gkl473 16849434 PMC1524920

[B58] Katayama S Tomaru Y Kasukawa T Waki K Nakanishi M Nakamura M Nishida H Yap CC Suzuki M Kawai J Antisense transcription in the mammalian transcriptome Science 2005 309 5740 1564 1566 16141073 10.1126/science.1112009

[B59] Werner A Carlile M Swan D What do natural antisense transcripts regulate? RNA Biol 2009 6 1 43 48 10.4161/rna.6.1.7568 19098462

[B60] Werner A Sayer JA Naturally occurring antisense RNA: function and mechanisms of action Curr Opin Nephrol Hypertens 2009 18 4 343 349 10.1097/MNH.0b013e32832cb982 19491676

[B61] Werner A Swan D What are natural antisense transcripts good for? Biochem Soc Trans 2010 38 4 1144 1149 10.1042/BST0381144 20659019 PMC4284956

[B62] Lapidot M Pilpel Y Genome-wide natural antisense transcription: coupling its regulation to its different regulatory mechanisms EMBO Rep 2006 7 12 1216 1222 10.1038/sj.embor.7400857 17139297 PMC1794690

[B63] Lasa I Toledo-Arana A Dobin A Villanueva M de los Mozos IR Vergara-Irigaray M Segura V Fagegaltier D Penades JR Valle J Genome-wide antisense transcription drives mRNA processing in bacteria Proc Natl Acad Sci USA 2011 108 50 20172 20177 10.1073/pnas.1113521108 22123973 PMC3250193

[B64] Faghihi MA Wahlestedt C Regulatory roles of natural antisense transcripts Nat Rev Mol Cell Biol 2009 10 9 637 643 10.1038/nrm2738 19638999 PMC2850559

[B65] Esterhuyse MM Linhart HG Kaufmann SH Can the battle against tuberculosis gain from epigenetic research? Trends Microbiol 2012 20 5 220 226 10.1016/j.tim.2012.03.002 22464289

[B66] Guida A Lindstadt C Maguire SL Ding C Higgins DG Corton NJ Berriman M Butler G Using RNA-seq to determine the transcriptional landscape and the hypoxic response of the pathogenic yeast *Candida parapsilosis* BMC Genomics 2011 12 628 10.1186/1471-2164-12-628 22192698 PMC3287387

[B67] Fasold M Binder H Estimating RNA-quality using GeneChip microarrays BMC Genomics 2012 13 1 186 10.1186/1471-2164-13-186 22583818 PMC3519671

[B68] Fang Z Martin JA Wang Z Statistical methods for identifying differentially expressed genes in RNA-Seq experiments Cell & bioscience 2012 2 1 26 10.1186/2045-3701-2-26 22849430 PMC3541212

[B69] Mortazavi A Williams BA McCue K Schaeffer L Wold B Mapping and quantifying mammalian transcriptomes by RNA-Seq Nat Methods 2008 5 7 621 628 10.1038/nmeth.1226 18516045 PMC13303166

[B70] Robinson MD McCarthy DJ Smyth GK edgeR: a Bioconductor package for differential expression analysis of digital gene expression data Bioinformatics 2010 26 1 139 140 10.1093/bioinformatics/btp616 19910308 PMC2796818

[B71] Smyth GK Linear models and empirical bayes methods for assessing differential expression in microarray experiments Stat Appl Genet Mol Biol 2004 3 Article3 16646809 10.2202/1544-6115.1027

[B72] Nookaew I Papini M Pornputtapong N Scalcinati G Fagerberg L Uhlen M Nielsen J A comprehensive comparison of RNA-seq-based transcriptome analysis from reads to differential gene expression and cross-comparison with microarrays: a case study in *Saccharomyces cerevisiae* Nucleic Acids Res 2012 40 20 10084 10097 10.1093/nar/gks804 22965124 PMC3488244

[B73] Ferrero E Biswas P Vettoretto K Ferrarini M Uguccioni M Piali L Leone BE Moser B Rugarli C Pardi R Macrophages exposed to *Mycobacterium tuberculosis* release chemokines able to recruit selected leucocyte subpopulations: focus on gammadelta cells Immunology 2003 108 3 365 374 10.1046/j.1365-2567.2003.01600.x 12603603 PMC1782907

[B74] Langmead B Trapnell C Pop M Salzberg SL Ultrafast and memory-efficient alignment of short DNA sequences to the human genome Genome Biol 2009 10 3 R25 10.1186/gb-2009-10-3-r25 19261174 PMC2690996

[B75] Trapnell C Pachter L Salzberg SL TopHat: discovering splice junctions with RNA-Seq Bioinformatics 2009 25 9 1105 1111 10.1093/bioinformatics/btp120 19289445 PMC2672628

[B76] R Development Core Team R: A language and environment for statistical computing 2011 Austria: R Foundation for Statistical Computing Vienna

[B77] Robinson MD Oshlack A A scaling normalization method for differential expression analysis of RNA-seq data Genome Biol 2010 11 3 R25 10.1186/gb-2010-11-3-r25 20196867 PMC2864565

[B78] Benjamini Y Hochberg Y Controlling the false discovery rate - a practical and powerful approach to multiple testing J Roy Stat Soc B Met 1995 57 1 289 300

[B79] Wilson CL Miller CJ Simpleaffy: a BioConductor package for Affymetrix Quality Control and data analysis Bioinformatics 2005 21 18 3683 3685 10.1093/bioinformatics/bti605 16076888

[B80] Wu ZJ Irizarry RA Gentleman R Martinez-Murillo F Spencer F A model-based background adjustment for oligonucleotide expression arrays J Am Stat Assoc 2004 99 468 909 917 10.1198/016214504000000683

[B81] Hochreiter S Clevert DA Obermayer K A new summarization method for affymetrix probe level data Bioinformatics 2006 22 8 943 949 10.1093/bioinformatics/btl033 16473874

[B82] Talloen W Clevert DA Hochreiter S Amaratunga D Bijnens L Kass S Gohlmann HWH I/NI-calls for the exclusion of non-informative genes: a highly effective filtering tool for microarray data Bioinformatics 2007 23 21 2897 2902 10.1093/bioinformatics/btm478 17921172

[B83] Durinck S Spellman PT Birney E Huber W Mapping identifiers for the integration of genomic datasets with the R/Bioconductor package biomaRt Nat Protoc 2009 4 8 1184 1191 10.1038/nprot.2009.97 19617889 PMC3159387

