# Supplementary figures and images for: Whole-transcriptome, high-throughput RNA sequence analysis of the bovine macrophage response to Mycobacterium bovis infection in vitro
Source: BMC Genomics. 2013 Apr 8;14:230. doi: 10.1186/1471-2164-14-230 (PMC3640917; doi:10.1186/1471-2164-14-230)

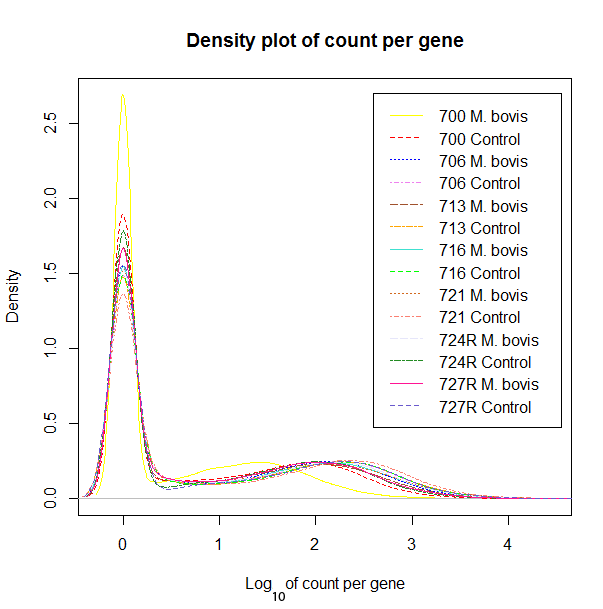

Supplement: Additional file 2: Figure S1 — Density plot of the distribution of reads per gene. Density plots of the number of sequence reads (in log10 space) per gene for each RNA-seq library sample. [file 1471-2164-14-230-S2.png]

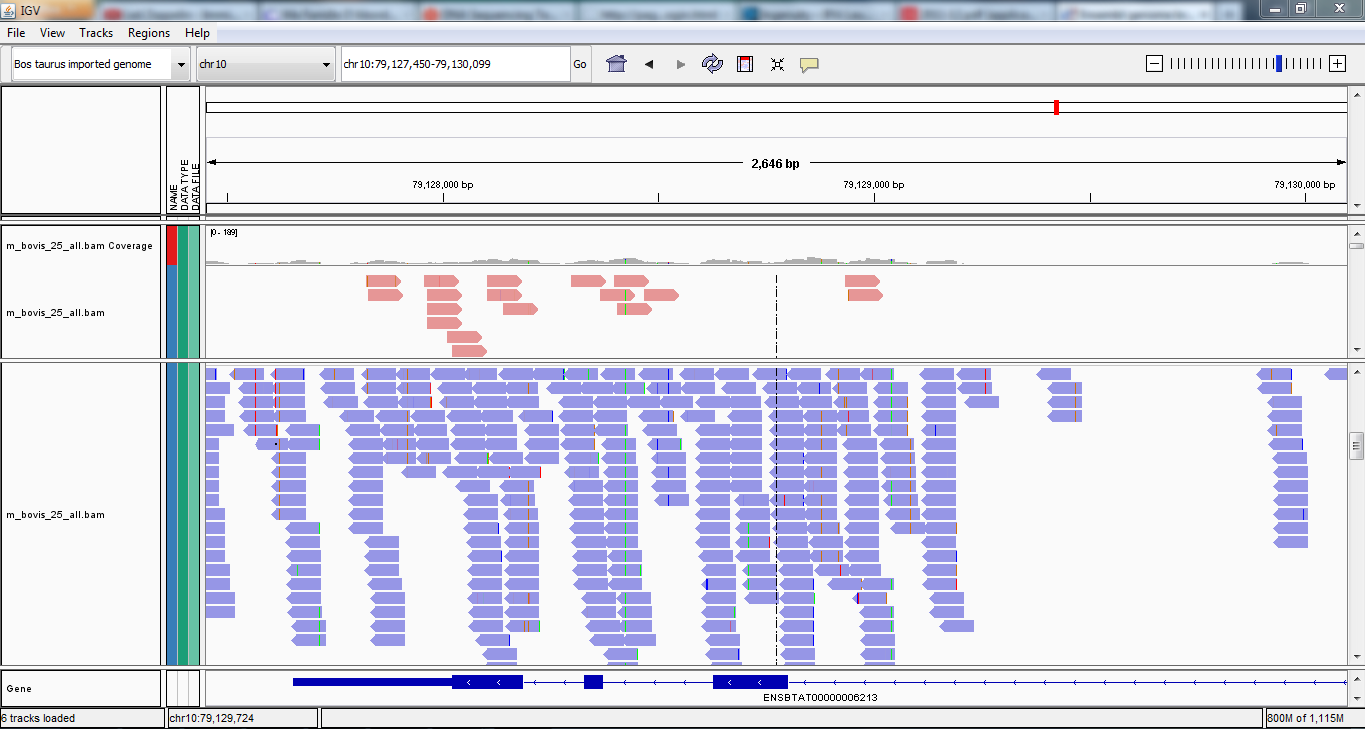

Supplement: Additional file 7: Figure S2 — Integrative Genomics Viewer (IGV) screen capture of reads mapping to SPTB gene. This figure shows the distribution of sense (represented in red) and antisense (represented in blue) strand reads that mapped to the 3′ end of spectrin, beta, erythrocytic gene (SPTB). [file 1471-2164-14-230-S7.png]

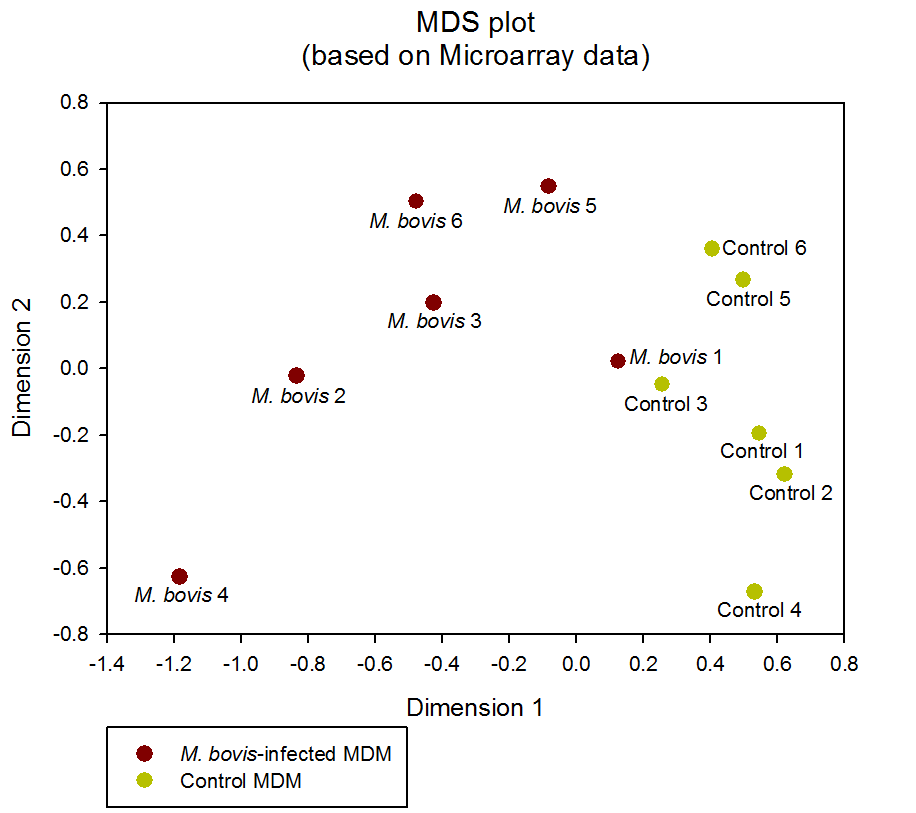

Supplement: Additional file 10: Figure S3 — Multi-dimensional scale plot of all M. bovis-infected and control samples based on Microarray data. Dimension 1 and dimension 2 separate all 12 samples based on the expression value of the 11,790 probes (based on microarray data only) that passed all data filtering criteria prior to differential gene expression analysis. [file 1471-2164-14-230-S10.png]

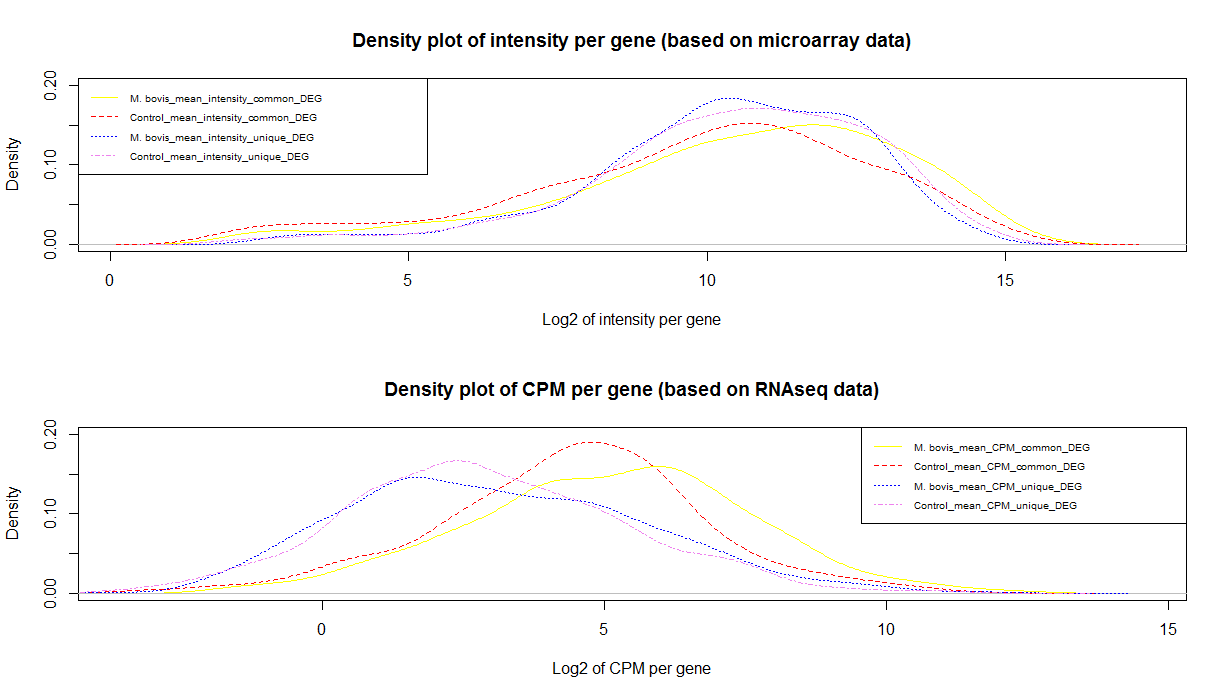

Supplement: Additional file 13: Figure S4 — Density plots of log2 mean CPM and log2 mean hybridisation intensities for differentially expressed genes unique and common to both platforms. This analysis was performed for each platform/treatment group. DEG, differentially expressed genes. [file 1471-2164-14-230-S13.png]
